# Models for estimating and projecting global, regional and national prevalence and disease burden of asthma: a systematic review

**DOI:** 10.7189/jogh.10.020409

**Published:** 2020-12

**Authors:** Mohammad Romel Bhuia, Md Atiqul Islam, Bright I Nwaru, Christopher J Weir, Aziz Sheikh

**Affiliations:** 1 Asthma UK Centre for Applied Research (AUKCAR), Usher Institute, The University of Edinburgh, Edinburgh, UK; 2 Department of Statistics, Shahjalal University of Science and Technology, Sylhet, Bangladesh; 3 Krefting Research Centre, Institute of Medicine, University of Gothenburg, Sweden; 4 Wallenberg Centre for Molecular and Translational Medicine, University of Gothenburg, Sweden; 5 Edinburgh Clinical Trials Unit, Centre for Population Health Sciences, Usher Institute, The University of Edinburgh, Edinburgh, UK

## Abstract

**Background:**

Statistical models are increasingly being used to estimate and project the prevalence and burden of asthma. Given substantial variations in these estimates, there is a need to critically assess the properties of these models and assess their transparency and reproducibility. We aimed to critically appraise the strengths, limitations and reproducibility of existing models for estimating and projecting the global, regional and national prevalence and burden of asthma.

**Methods:**

We undertook a systematic review, which involved searching Medline, Embase, World Health Organization Library and Information Services (WHOLIS) and Web of Science from 1980 to 2017 for modelling studies. Two reviewers independently assessed the eligibility of studies for inclusion and then assessed their strengths, limitations and reproducibility using pre-defined quality criteria. Data were descriptively and narratively synthesised.

**Results:**

We identified 108 eligible studies, which employed a total of 51 models: 42 models were used to derive national level estimates, two models for regional estimates, four models for global and regional estimates and three models for global, regional and national estimates. Ten models were used to estimate the prevalence of asthma, 27 models estimated the burden of asthma – including, health care service utilisation, disability-adjusted life years, mortality and direct and indirect costs of asthma – and 14 models estimated both the prevalence and burden of asthma. Logistic and linear regression models were most widely used for national estimates. Different versions of the DisMod-MR- Bayesian meta-regression models and Cause Of Death Ensemble model (CODEm) were predominantly used for global, regional and national estimates. Most models suffered from a number of methodological limitations – in particular, poor reporting, insufficient quality and lack of reproducibility.

**Conclusions:**

Whilst global, regional and national estimates of asthma prevalence and burden continue to inform health policy and investment decisions on asthma, most models used to derive these estimates lack the required reproducibility. There is a need for better-constructed models for estimating and projecting the prevalence and disease burden of asthma and a related need for better reporting of models, and making data and code available to facilitate replication.

Resources are limited and health system planners need to make investment decisions based on available evidence of disease prevalence, associated morbidity and health care utilisation, mortality and costs, how these are likely to have the potential to change over time and their amenability to interventions [1-8]. Modelling studies have the potential to inform such important deliberations [1,9-11], hence, they have been prioritised by inter-governmental organisations such as the World Health Organization (WHO) and funders such as Bill and Melinda Gates Foundation [3,5,8,12-17]. The Institute for Health Metrics and Evaluation (IHME) [18] and The Child Health Epidemiology Reference Group (CHERG) [19] are two prominent examples of initiatives that have extensively used modelling approaches to generate estimates of disease epidemiology and morbidity.

Focusing specifically on estimates of asthma prevalence and burden, we observe that varying estimates have been reported in the published literature at national, regional and global levels, which are not consistent over the years (as shown in **Table 1**). Given the range of models used to estimate prevalence and burden of asthma, it is a challenging task to determine an appropriate model that can be applied in a new data set. Furthermore, there is a lack of a robust critical evidence base to help guide decisions on which model(s) are most appropriate for different contexts. There is, therefore, a need for a systematic appraisal of the merits and limitations of available models. In this study, we sought to systematically describe and critique existing models for prevalence and burden of asthma in relation to their strengths, limitations and reproducibility.

**Table 1:** Global prevalence and burden (mortality) of asthma

Study	Year	Prevalence in thousands (uncertainty interval)	Burden: mortality in thousands (uncertainty interval)
GBD [12,13]	2017	272 677 (242 295-304 699)	495 (338-641)
GBD [3,17]	2016	339 440 (319 582-360 796)	420 (338.8-517.7)
GBD [14]	2015	358 198 (323 134-393 466)	397 (363-439)
GBD [5,8]	2013	241 695 (238 151-245 465)	489 (397.7-676.8)
GBD [1,9]	2010	334 247 (Not available)	345.7 (282.6–529.1)
WHO [20]	2004	234 900 (Not available)	287 (Not available)
			

*GBD – Global Burden of Disease, WHO – World Health Organization

## METHODS

### Protocol

We described and documented the methods employed in this systematic review in detail in a previously published protocol [21] and therefore confine ourselves here to a summary of the methods employed.

### Eligibility criteria

We included any study that applied models for estimating and projecting prevalence or disease burden of asthma. We included original research and review articles, including systematic reviews, meta-analyses and meta-syntheses of observational studies in human populations of any age and sex. We included research articles from any country and any setting (urban/rural) and published in any language. Our outcomes of interest were the prevalence of asthma and different components of the disease burden of asthma. The components of disease burden were direct and indirect costs of asthma (costs due to primary care utilisation, hospitalisation, ambulatory care, emergency visit, drug cost, absenteeism, presentism), disability-adjusted life years (DALYs), years lived with disability (YLDs), years of life lost (YLLs), potential years of life lost, healthy years of life lost, active life expectancy, disability-free life expectancy, disability-adjusted life expectancy, and healthy life expectancy (HALE).

### Information sources

We identified relevant published and unpublished studies through searching electronic databases, hand-searching of pertinent journals and checking reference lists of all the eligible papers for studies published between January 1980 and September 2017. The following electronic databases were searched: Medline, Embase, World Health Organization Library and Information Services (WHOLIS library catalogue of books and reports) and Web of Science Core Collection. Journals that were hand-searched included The Lancet, BMJ (1980-2017), European Respiratory Journal (1988-2017), Lancet Respiratory Medicine (2013-2017), Lancet Global Health (2013-2017) and Journal of Global Health (2011-2017). We ran the last searches on 16 September 2017. We included papers published in any language and translated the papers that were not in English with the help of fluent or native speakers.

### Search strategy

Comprehensive search strategies were developed for all the aforementioned databases in consultation with a senior medical librarian at The University of Edinburgh to identify both published and unpublished (grey literature) primary studies as well as reviews. The search terms used included but were not limited to asthma, wheeze, epidemiology, prevalence, burden, morbidity, mortality, DALY, QALY, HALE, primary health care, emergency service, hospitalisation, absenteeism, cost of illness, model, estimate, projection. The detailed search strategies used to search each database are given in Appendix S1 in the **Online Supplementary Document**.

### Study selection

Two reviewers independently checked and screened the titles and abstracts of identified articles against the inclusion criteria. Full-text copies of potentially eligible studies were obtained and assessed by two independent reviewers (MRB and MAI) on the basis of the inclusion criteria. Discussion between the two reviewers resolved the majority of discrepancies; a third reviewer (BIN) arbitrated in the case of any disagreements.

### Data collection process

We developed a data extraction form and used it to extract relevant data from included studies. The data extraction form was piloted on 10 included studies and was then refined accordingly prior to full use in the review. Data extraction was performed independently by two reviewers (MRB and MAI). Any discrepancies in data extraction were resolved through discussion between the reviewers, with arbitration by a third reviewer (BIN) if a decision could not be reached.

### Data items

The following data items were extracted from each paper: 1) study identification (authors’ name, study/publication year, title); 2) aims and methods of the study (context of the study; country or region; study outcomes; burden type; case definitions; type of estimation; data and study population: data sources, age, sex, study area; estimation/study period; study design; data type; response variables; and data level); 3) model information (model name; model purpose; model structure; appropriateness of the model; model assumptions; model building; model fitting; model diagnosis; goodness-of-fit; robustness; missing data; uncertainty estimation; validation; sensitivity analysis; adequate model presentation; adequate reporting of estimates; reproducibility: availability of input data, computer coding and model fitting manual).

### Summary measures

As this review mainly focused on the properties of models rather than quantitative measures, we did not perform any quantitative synthesis of the data.

### Methods of analysis

We produced a tabular summary of the data to summarise the overall evidence. Descriptive statistics and graphical presentation were used to summarise the results. A detailed critical narrative synthesis of the models was undertaken regarding their strengths, limitations and reproducibility.

### Quality appraisal of the included models

To our knowledge, there is no existing quality appraisal tool to assess the quality of the components of models for estimating the prevalence and burden of diseases. We therefore developed our own model evaluation checklist by adapting relevant sections from pertinent critical appraisal checklists [22,23], reporting guidelines [24] and other guidelines for good practice in modelling studies [25-28]. Prior to finalising this checklist, we consulted three experts (two medical statisticians and an epidemiologist) in modelling studies.

### Assessing strengths, limitations and reproducibility of the included models

**Figure 1:**
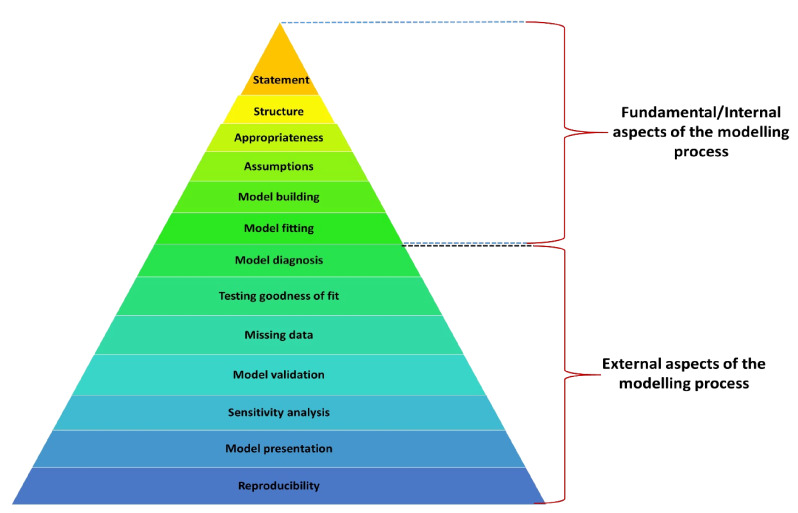
Checklist for assessing the quality of models.

Our checklist consists of 13 quality criteria that a model should possess. We present the checklist by a pyramid in **Figure 1** in terms of the hierarchy of the model quality criteria. Table S1 in the **Online Supplementary Document** provides a description of these criteria. We classified the model quality criteria into two groups: 1) fundamental or internal aspects of the modelling process; and 2) external aspects. The fundamental or internal aspects are: model statement, model structure, model appropriateness, model assumptions, model building and model fitting. The external aspects are: model diagnosis, testing goodness-of-fit, addressing missing data, model validation, sensitivity analysis, adequate presentation and reporting of model to ensure reproducibility.

### Reporting

This systematic review is reported following the guidelines of the Preferred Reporting Items for Systematic review and Meta Analysis (PRISMA) checklist [29].

## RESULTS

### Study selection

**Figure 2:**
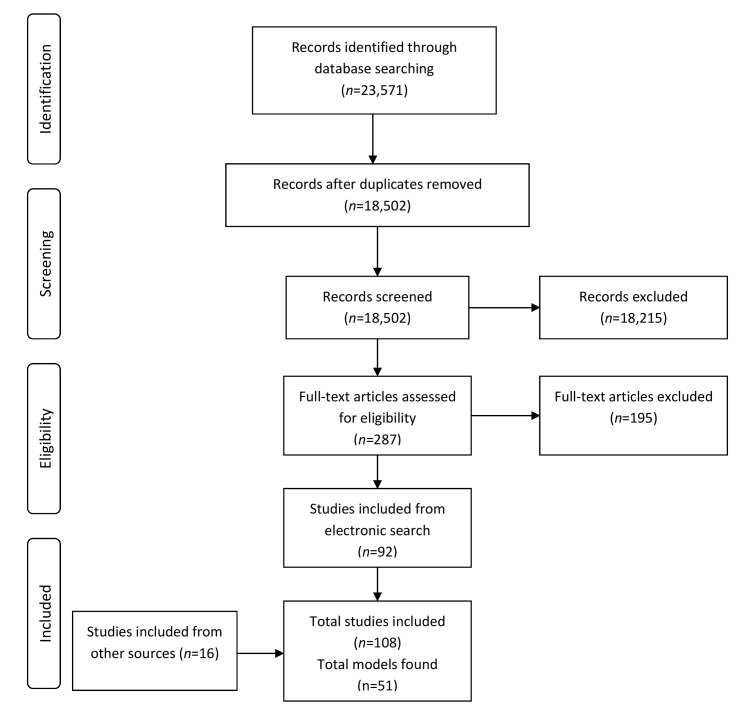
PRISMA flow diagram of selected papers.

Our electronic search of the databases yielded a total of 23 571 references. After removing duplicates, 18 502 study titles and abstracts were screened against our inclusion criteria. Among these, 287 full-text articles were assessed in detail. Finally, 92 papers met the inclusion criteria. An additional 16 papers identified from hand-searching of journals and scrutinising reference lists of included studies met the inclusion criteria. Therefore, a total of 108 studies were included in the systematic review. The PRISMA flow diagram of the selected papers is presented in **Figure 2**. We included five non-English papers: two Dutch papers, one German paper, one French paper and one Spanish paper.

### Study characteristics

**Figure 3:**
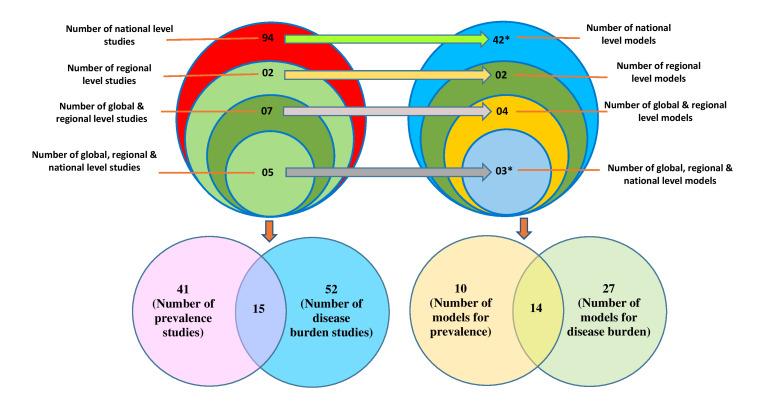
Distribution of included studies and models. *One model (linear regression model) was used in both national-level and global-and-regional-level studies which we counted as national level model due to its high uses in national level studies; and one model (CODEm) was used in both global-and-regional-level and global-regional-and national-level studies which we counted as global-regional-and national-level model due to its high uses in global-regional-and national-level studies.

We identified 94 studies that estimated the prevalence and burden of asthma at a national level; two at a regional level; seven at both global and regional levels; and five at global, regional and national levels (**Figure 3**). Among the 108 included studies, 41 studies estimated the prevalence of asthma, 52 studies estimated the burden of asthma and 15 studies estimated both prevalence and burden of asthma (**Figure 3**). Most of the included studies originated from Europe (n = 33, 30.6%); followed by North America (n = 29, 26.9%), worldwide or multi-country (n = 19, 17.6%), Asia (n = 15, 13.9%), South America (n = 7, 6.5%) and Australia (n = 4, 3.7%); and only one study was from Africa (**Table 2**).

**Table 2:** Distribution of included studies by region

Region	Number of studies (%)
Africa	1 (1.0)
Asia	15 (13.9)
Australia	4 (3.7)
Europe	33 (30.6)
North America	29 (26.9)
South America	7 (6.5)
Worldwide or multi-country	19 (17.6)
**Total**	**108 (100.0)**

### Models for estimating and projecting prevalence and burden of asthma

A total of 51 models were used in the 108 included studies: 42 models were used to derive national level estimates (41 national level models plus one model common with global-and-regional level); two models were used to derive regional level estimates; four were used for global and regional estimates; and three models were used to derive global, regional and national estimates (**Figure 3**). Among these 51 models, ten models were used to estimate the prevalence of asthma, 27 models were used to estimate the burden of asthma, and 14 models were used to estimate both the prevalence and burden of asthma (**Figure 3**). Distribution of all the models is presented by study level and type of measurement in **Table 3**.

**Table 3:** Distribution of models by study level and type of measurement

Study level	Type of measurement		
**Prevalence**	**Burden**	**Both prevalence and burden**
**National**	1. Meta-analysis: random effect model	1. Two-part models	1. Logistic regression model
	2. Logistic regression model with regression splines/restricted cubic splines	2. Generalised linear models with gamma distribution and logarithmic link function	2. Linear regression model*
	3. Exponential regression model	3. Log transformed linear model	3. Poisson regression model
	4. General linear predictive model	4. LOESS (locally weighted regression) model	4. Negative binomial regression model
	5. Hierarchical logistic regressions model	5. Bootstrapped prevalence-based cost of illness model	5. Generalised estimating equations (GEE)
	6. Survey weighted logistic regression model	6. Box-Jenkins regression-ARIMA model	6. Generalised linear models
		7. Conditional Autoregressive (CAR) model	7. Generalised linear mixed effect model
		8. Cost assessment model	8. Computer simulation model
		9. Economic model	9. Double exponential smoothing model
		10. Exchangeable (EX) Model – Poisson-Gamma Model	10. Epidemiological model based on a dynamic multi-state lifetable
		11. First degree homogeneous Markov model	11. RIVM Chronic disease model
		12. Generalised additive model (GAM)	
		13. Heckman selection model	
		14. Joinpoint regression model	
		15. Log-linear autoregression model	
		16. Log-linear regression model	
		17. Machine learning based prediction model	
		18. Multiplicative models for rates (Beslow and Day method)	
		19. Multivariate regression model with weighted least squares	
		20. Polynomial regression model	
		21. Quadratic regression model	
		22. Quantile regression model	
		23. Seasonal autoregressive integrated moving average (SARIMA) model	
		24. Weighted linear regression model	
		25. Zero-inflated negative binomial regression model	
**Regional**	1. Nonlinear exponential regression model	-	-
	2. Meta-analysis: random effects Bayesian model		
**Global and regional**	1. DisMod-MR	1. Cause Of Death Ensemble modeling (CODEm)†	1. DisMod
		2. Cause-of-death modeling (CodMod)	2. DisMod II
		3. Linear regression model*	
**Global, regional and national**	1. DisMod-MR 2.0	1. Cause Of Death Ensemble modeling (CODEm)†	1. DisMod-MR 2.1

*Linear regression model was used in both national-level and global-and-regional-level studies which we counted as national level model due to its high uses in national level studies.

†CODEm was used in both global-and-regional-level and global-regional-and national-level studies which we counted as global-regional-and national-level model due to its high uses in global-regional-and national-level studies.

### Outcome measures and statistical methods used by the included studies

A tabular summary of the data is presented in Table S2 in the **Online Supplementary Document** to summarise the overall evidence. The included studies mainly used models for deriving annual estimates, trends, changes of estimates over a period, projections, predicted estimates and forecasted estimates of prevalence and various components of the disease burden of asthma. In addition to estimates of prevalence, the components of disease burden that were estimated were mortality; health care service utilisation (health care provider visit, family practitioner visits, specialist visits, emergency room visits/emergency department visits, hospitalisation/hospital admission, re-admission); productivity loss (due to absenteeism, presenteeism and overall work impairment); cost of asthma – direct medical expenditure (costs of physician office visits, emergency department visits, outpatient visits, inpatient visits, medications, and other medical visits, asthma exacerbations and readmissions), indirect costs (costs of absenteeism, costs of parents’ productivity loss and work-time loss, children’s loss of lifetime earnings because of premature death), societal costs; incidence; asthma exacerbation; YLDs; and quality of life.

**Figure 4:**
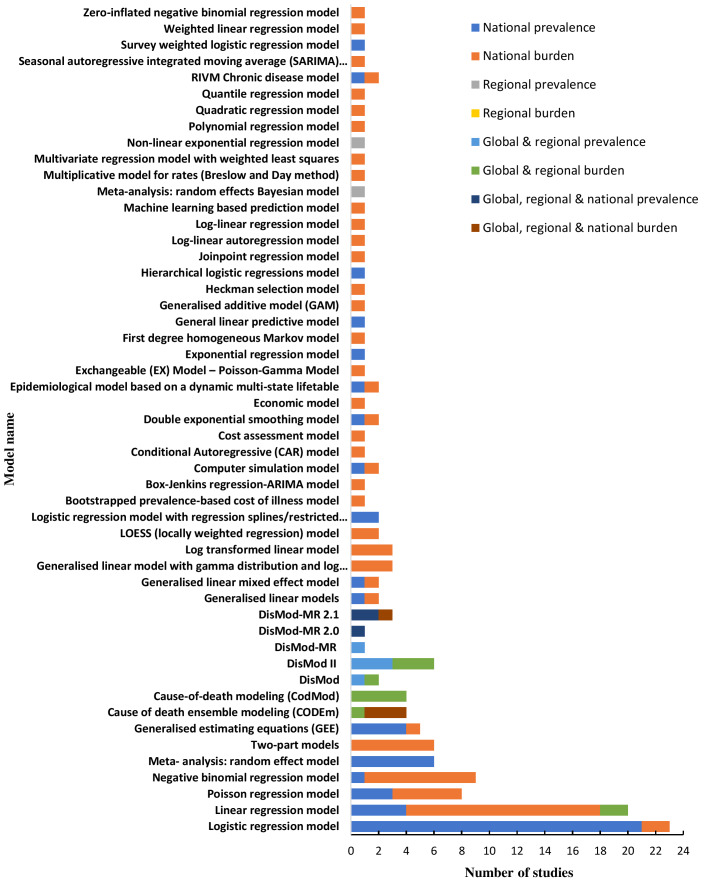
Frequencies of uses of each model by type of study. NB. Sum of the frequencies of uses of a model may not be equal to the number of studies used that model, because many studies used more than one model and some studies used same model for estimating both prevalence and more than one component of burden.

The frequencies of uses of each model by type of study are shown in **Figure 4**. Logistic regression modelling was the most commonly used approach for estimating the national prevalence of asthma, which was used in 21 studies [30-50] (out of 46 studies that estimated national prevalence). This was followed by linear regression, which was used in 14 studies [51-64] (out of 57 studies) to estimate and project the national burden of asthma. DisMod, DisMod II and cause of death modelling (CodMod) were used to estimate global and regional prevalence and burden of asthma in four studies [10,20,65,66] (out of seven studies). Different versions of DisMod-MR-Bayesian meta-regression models (DisMod-MR, DisMod-MR 2.0 and DisMod-MR 2.1) and Cause Of Death Ensemble modelling (CODEm - mixed effects linear/nonlinear models and/or spatial-temporal Gaussian process regression models) were used in all the five studies [3,5,8,14,17] for estimating the global, regional and national prevalence and burden of asthma.

### Strengths, limitations and reproducibility of the included models

**Figure 5:**
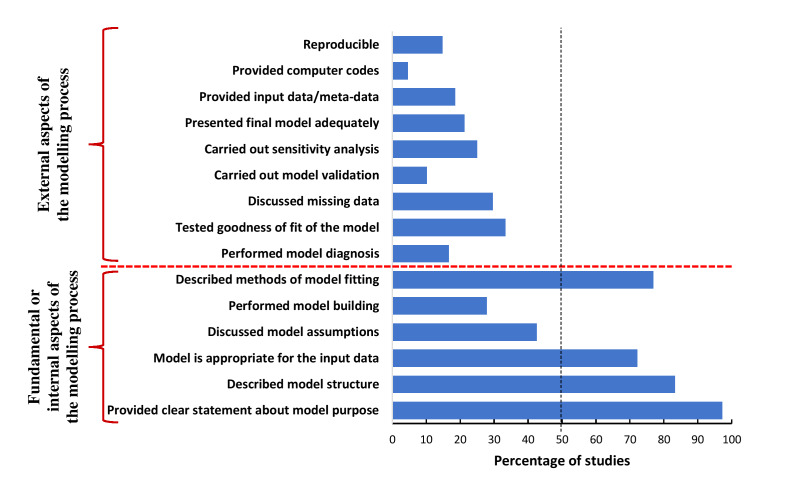
Percentage of studies fulfilled each model quality criteria.

**Figure 5** presents the results emanating from the application of the checklist we developed to appraise the quality of models.

#### Strengths of the models

More than half of the studies fulfilled four of the six internal model quality criteria. One hundred and five (97.2%) studies [1,3,5,8-10,14,17,20,30,32,33,35-127] provided a clear statement about the questions that the models aimed to answer. Four in five studies (n = 90; 83.3%) [1,3,5,8,10,14,17,20,30,33,34,36-44,46-51,53-55,57,59-70,72,73,75-78,81-84,87,89-111,113-120,122-127] explicitly described the structure of the models, and nearly three-quarters of studies (n = 78; 72.2%) [1,3,5,8-10,14,17,20,30,33-44,46-50,65-67,70-72,75-77,81,82,84,85,87-94,96-114,116-120,123-128] used appropriate modelling tools to deal with the nature, distribution and type of input data. Adequate description of the process of model fitting was provided by 83 (76.9%) studies [1,3,5,8-10,14,17,20,34-44,46-51,53-55,57,60-66,68-70,72,76,78,81-84,87,89-111,113-119,122,124-127] (**Figure 5**).

#### Limitations of the models

Most studies failed to fulfil two internal model quality criteria and all the external criteria. Sixty-two (57.4%) studies [30,32-34,36-38,40,41,44-59,61-64,67,68,71,73,74,79,81,83,85,87,88,92,93,95,96,98-101,104,106,109-115,117,122,127-129] did not provide any information about the model assumptions, while, only 30 (27.8%) studies [1,3,8,14,33,36,38,44,50,55,57,67,70,72,75-77,82,84,90-92,94,96,97,100,103,110,116,124] performed model building to select necessary variables for the models. The model diagnosis (model adequacy checking) was performed by 18 (16.7%) studies [49,68,69,72,75,76,82,86,89-91,93,97,101,103,105,107,118], whereas 36 (33.3%) studies [1,3,5,8,14,46,49-51,54-56,60,61,63,64,67,69,72,81,82,84,89-91,94,96,97,103,107,110,117-119,122,124] tested and reported goodness-of-fit of the models. Very few studies discussed missing data (n = 32; 29.6%) [1,3,5,8-10,14,17,20,32,33,44,46,48,58,65,66,70,76,78,85,86,89,92,94,97,109,114,117,118,124,126], carried out model validation (n = 11; 10.2%) [1,3,5,8,14,41,80,84,96,97,123], performed sensitivity analysis (n = 27; 25%) [1,10,14,20,41,43,45,55,65,72,75,76,78-80,84,88,93,104,108,112,118-121,123,124] and presented final model adequately (n = 23; 21.3%) [40,51,53-56,62,69,75,81,84,89,90,93-95,97,99,101,106,107,117,124] (**Figure 5**).

### Reproducibility

We found only 20 (18.5%) studies [3,14,17,49,51,52,54,62,69,81,90,93,98,99,101,104,110,114,117,122] that provided final input data or meta-data. Very few studies (n = 5; 4.6%) [3,14,17,90,117] provided the computer code used to fit the models, and very few models (n = 16; 14.8%) [49,51,54,62,69,81,90,93,98,99,101,104,110,114,117,122] were therefore judged to be reproducible (**Figure 5**).

Further analysis, assessing whether the included studies adhered the new Guidelines for Accurate and Transparent Health Estimates Reporting (GATHER) [24] showed that most of the included studies (n = 105; 97%) did not adhered the GATHER guidelines to report their derived estimates of prevalence and burden of asthma; nevertheless, 82% (n = 88) of these studies were published before the publication of GATHER guidelines. Among the 20 studies, that were published after the availability of GATHER guidelines, only three studies (15%) [3,14,18] adhered these guidelines to report their estimates.

## DISCUSSION

### Statement of principal findings

This systematic review of the international literature has found that a variety of models are used to estimate disease prevalence and burden; most models, however, suffer from methodological limitations, in particular, lack of reproducibility and sub-optimal reporting.

Almost all the studies provided a clear statement about the questions that the models aimed to answer. The majority of the studies described the structure of the models explicitly; mentioned the methods used to fit the models or used to estimate model parameters; and applied appropriate modelling tools to deal with the nature, distribution and type of input data. These were the strengths of the models. However, these models had substantial limitations. We observed a lack of clarity in reporting the models. The majority of the studies did not provide any information on the model assumptions, methods of model building, methods of model diagnosis, methods of handling missing data, model validation and sensitivity analysis. In addition, many studies did not adequately present the final model parameter estimates, standard errors and confidence intervals. Moreover, most of the models could not be reproduced as the studies did not provide input data (or alternatively meta-data, synthetic data or simulated data if input data could not be published due to confidentiality), computer coding, a model fitting manual or complete information about the process of model formulation.

### Strengths and limitations

The main strengths of this review include the comprehensive search strategy employed, the use of established methodology (two independent reviewers for screening, full-text assessment and data extraction) and the inclusion of studies from all over the world including studies published in any language. Critical appraisal of identified models and expert involvement in developing model assessment tools further strengthened the credibility of this review. However, this review has a number of potential limitations. As with any systematic review, we may have missed some studies. Although risk factor based models or association models are particularly essential to assess determinants of a disease, we did not include studies that used models to estimate risk factors or to assess association rather than estimating prevalence and disease burden using model. The use of a self-developed customised model evaluation tools, due to the lack of appropriate critical appraisal checklist, may be considered to be a further limitation of this review. There is a need for the development of an internationally standard tool that will be used for the purpose.

### Interpretation in the context of the wider literature

This systematic review is the first to synthesise models for prevalence and burden of asthma in the context of national, regional and global estimates and projections. Previous systematic reviews on disease modelling studies were mainly undertaken on other domains, such as prediction models [130-139], economic models [140] and decision analytic models [141-143]. Most of the findings of our review are in line with these previous reviews, suggesting that inadequate model development and poor reporting quality are the key issues in modelling studies that chiefly affect the quality of model-derived estimates and hinder the assessment of the usability of the models. A systematic review on projection models for prevalence and burden of chronic obstructive pulmonary disease (COPD) [144] argued that there was no consensus on the best model structure as models varied depending on the purpose and contexts of modelling. Another review on coronary heart disease policy models [25] emphasised introducing standard reporting guidelines to improve the reporting quality of models.

### Implications for policy, practice and future research

#### Implication for asthma policy

Existing estimates are heavily reliant on modelling studies due to lack of data on direct measurements of asthma cases in many countries and regions. Whilst these modelling studies have advanced better understanding and appreciation of the burden of different diseases, the lack of reproducibility of the models, as highlighted in this review, requires concerted effort from researchers and decision makers to set in place platforms that will ensure that estimates of disease burden produced can be reproduced. Policymakers should thus be aware of the transparency of modelling processes and the reliability of the input data when making decisions on the basis of these model-based estimates.

#### Implications for model developers

The findings of this review suggest that models should be carefully designed to incorporate all the necessary methodological components required to develop a robust model, including an explicit statement about the purpose and structure of the model; statement of necessary model assumptions; variable selection applying appropriate techniques; model diagnostic accuracy checking; assessing goodness-of-fit; addressing missing data by applying suitable techniques; applying optimum methods of parameter estimation; carrying out sensitivity analysis; and performing both internal and external model validation. Besides, a highly complex model usually lacks understanding, usability, reproducibility and, hence, credibility. While publishing models, sufficient information about the complete modelling process, therefore, should be reported to facilitate its understanding and usability for non-technical audiences. For example, a model development manual should be made publicly available, including input data and necessary computer code, to describe the step-by-step process of model development with illustrative examples. Although the perspectives of this review are prevalence and disease burden of asthma, these recommendations also apply to the modelling prevalence and burden of other chronic diseases.

#### Implication for future research

Future research could potentially be undertaken to develop consensus guidelines for developing or fitting and reporting models for prevalence and burden of diseases. Moreover, developing a critical appraisal checklist for assessing the quality of models for prevalence and burden of diseases is another key area for future research.

Of the available approaches, we found the Bayesian meta-regression method- DisMod-MR, DisMod-MR 2.0, DisMod-MR 2.1 and CODEm [1,3,5,8,9,14,17] modelling tools faired best as they fulfilled most of our model quality criteria and were specially designed to deal with the diversity of data (multiple sources and designs) [11,145] needed to derive national, regional and global, level estimates. However, these modelling methods lack usability for the general user because of unavailability of sufficient technical detail and customised packages in standard statistical software such as R, SAS, and STATA. Therefore, more work needs to be done with these models to improve their usability. Moreover, DisMod-MR and CODEm are used as generic models by the Global Burden of Disease (GBD) collaborators to derive health estimates for numerous diseases and injuries. Therefore, the potential added value of well-constructed asthma-specific models should be considered.

## Conclusions

Amidst data types and their sources, modelling remains indispensable for estimating the prevalence and burden of disease. This evidence synthesis has shown that existing models that have been applied to estimate the prevalence and burden of asthma suffer from methodological limitations, in particular, suboptimal reporting and lack of reproducibility. There is a need to enhance the reportage of models used for estimating and projecting the prevalence and disease burden of asthma and making data and code available to facilitate replication. Moreover, there is also a need for developing better-constructed asthma-specific models in an attempt to produce more accurate and consistent estimates. In the interim, we suggest using Bayesian meta-regression models and cause of death ensemble models for estimating national, regional and global prevalence and burden of asthma, and Box-Jenkins regression- autoregressive integrated moving average (ARIMA) model to make projections in relation to these estimates. We also suggest to validate the Bayesian meta-regression models against their alternative frequentist or classical models to check which modelling approaches generate better estimates of prevalence and burden of asthma than the others.

## Additional material

Online Supplementary Document
