# Supplementary material for: Models for estimating and projecting global, regional and national prevalence and disease burden of asthma: a systematic review
Source: J Glob Health. 2020 Dec 30;10(2):020409. doi: 10.7189/jogh.10.020409 (PMC7774028; doi:10.7189/jogh.10.020409)
Supplement: Online Supplementary Document [file jogh-10-020409-s001.pdf]

Online Supplementary Document

Models for estimating and projecting global, regional and national prevalence and disease burden of asthma: a systematic review

Appendix S1: Search strategy

MEDLINE

1. exp Asthma/
2. Respiratory Sounds/
3. wheez$.mp.
4. Bronchial Spasm/
5. bronchospas$.mp.
6. (bronch$ adj3 spasm$).mp.
7. bronchoconstrict$.mp.
8. asthma$.mp.
9. (antiasthma$ or anti-asthma$).mp.
10. exp Bronchoconstriction/
11. (bronch$ adj3 constrict$).mp.
12. Bronchial Hyperreactivity/
13. Respiratory Hypersensitivity/
14. ((bronchial$ or respiratory or airway$ or lung$) adj3 (hypersensitiv$ or hyperreactiv$ or allerg$ or insufficiency)).mp.
15. or/1-14
16. Epidemiology/
17. morbidity/ or incidence/ or prevalence/ or mortality/
18. (disability-adjusted life years or disability adjusted life years or DALY$).mp.
19. Quality-Adjusted Life Years/ or (quality adjusted life years or QALY$).mp.
20. years lived with disability.mp.
21. years life lost.mp.
22. potential years of life lost.mp.
23. healthy years of life lost.mp.
24. active life expectancy.mp.
25. (disability-free life expectancy or disability free life expectancy).mp.

26. (disability-adjusted life expectancy or disability adjusted life expectancy).mp.
27. (Healthy life expectancy or HALE).mp.
28. (quality adjusted life expectancy or quality-adjusted life expectancy).mp.
29. vital statistics/ or life expectancy/ or life tables/
30. "costs and cost analysis"/ or "cost of illness"/ or health care costs/ or drug costs/ or hospital costs/ or health expenditures/
31. Hospitalization/ or hospitalisation.mp.
32. Primary Health Care/ or Ambulatory Care/ or Emergency Service, Hospital/ or absentee$.mp.
33. burden$.mp.
34. or/16-33
35. model$.mp. or exp models, statistical/ or exp regression analysis/ or odds ratio/ or monte carlo method/ or markov chains/ or Markov.mp.
36. (estimat$ or projection$ or projecting).ti.
37. exp Forecasting/
38. or/35-37
39. 15 and 34 and 38

EMBASE

1. exp asthma/
2. asthma$.mp.
3. (antiasthma$ or anti-asthma$).mp.
4. abnormal respiratory sound/
5. wheez$.mp.
6. bronchospasm/
7. bronchospas$.mp.
8. (bronch$ adj3 spasm$).mp.
9. bronchoconstrict$.mp.
10. bronchoconstriction/
11. (bronch$ adj3 constrict$).mp.
12. bronchus hyperreactivity/
13. respiratory tract allergy/
14. ((bronchial$ or respiratory or airway$ or lung$) adj3 (hypersensitiv$ or hyperreactiv$ or allerg$ or insufficiency)).mp.

15. or/1-14
16. epidemiology/
17. Prevalence/ or Incidence/ or Morbidity/ or Mortality/
18. burden$.mp.
19. (disability-adjusted life years or disability adjusted life years or DALY$).mp.
20. quality adjusted life year/ or (quality-djusted life years or QALY$).mp.
21. years lived with disability.mp.
22. years life lost.mp.
23. potential years of life lost.mp.
24. healthy years of life lost.mp.
25. Vital statistics/ or Life expectancy/ or Life tables/
26. active life expectancy.mp.
27. (disability-free life expectancy or disability free life expectancy).mp.
28. (disability-adjusted life expectancy or disability adjusted life expectancy).mp.
29. (Healthy life expectancy or HALE).mp.
30. (quality adjusted life expectancy or quality-adjusted life expectancy).mp.
31. "health care cost"/ or "drug cost"/ or "hospital cost"/ or "hospitalization cost"/ or "cost"/ or "cost of illness"/
32. hospitalization/ or Hospitalisation.mp.
33. primary health care/ or medical care/ or emergency care/ or emergency treatment/ or emergency health service/ or ambulatory care/ or absenteeism/
34. or/16-33
35. model/ or loglinear model/ or population model/ or mathematical model/ or proportional hazards model/ or statistical model/ or stochastic model/ or markov chain/ or Markov.mp.
36. (estimat$ or projection$ or projecting).mp.
37. "prediction and forecasting"/
38. or/35-37
39. 15 and 34 and 38

Web of Science Core Collection

#4	#3 AND #2 AND #1<br>Refined by: [excluding] WEB OF SCIENCE CATEGORIES: (METEOROLOGY ATMOSPHERIC SCIENCES OR VETERINARY SCIENCES OR ENGINEERING BIOMEDICAL OR PLANT SCIENCES OR FOOD SCIENCE TECHNOLOGY OR NEUROSCIENCES OR COMPUTER SCIENCE INTERDISCIPLINARY APPLICATIONS OR CONSTRUCTION BUILDING TECHNOLOGY OR SUBSTANCE ABUSE OR REHABILITATION OR COMPUTER SCIENCE INFORMATION SYSTEMS OR PSYCHOLOGY DEVELOPMENTAL OR MATHEMATICS INTERDISCIPLINARY APPLICATIONS OR ANESTHESIOLOGY OR ENGINEERING ELECTRICAL ELECTRONIC OR REPRODUCTIVE BIOLOGY OR TRANSPLANTATION OR INFECTIOUS DISEASES OR BIOTECHNOLOGY APPLIED MICROBIOLOGY OR CHEMISTRY MULTIDISCIPLINARY OR PSYCHIATRY OR PARASITOLOGY OR BIOCHEMICAL RESEARCH METHODS OR BIOCHEMISTRY MOLECULAR BIOLOGY OR ORTHOPEDICS OR PERIPHERAL VASCULAR DISEASE OR ECOLOGY OR EDUCATION SCIENTIFIC DISCIPLINES OR WOMEN S STUDIES OR OBSTETRICS GYNECOLOGY OR FAMILY STUDIES OR CLINICAL NEUROLOGY OR RADIOLOGY NUCLEAR MEDICINE MEDICAL IMAGING OR CELL BIOLOGY OR INFORMATION SCIENCE LIBRARY SCIENCE OR BIOPHYSICS OR ENGINEERING MECHANICAL OR DENTISTRY ORAL SURGERY MEDICINE OR BIODIVERSITY CONSERVATION OR NUTRITION DIETETICS OR UROLOGY NEPHROLOGY OR BEHAVIORAL SCIENCES OR SURGERY OR SPORT SCIENCES OR ENGINEERING CIVIL OR ENGINEERING ENVIRONMENTAL OR VIROLOGY OR CHEMISTRY ANALYTICAL OR OPHTHALMOLOGY OR SOCIAL SCIENCES MATHEMATICAL METHODS OR MICROBIOLOGY OR COMPUTER SCIENCE THEORY METHODS OR GASTROENTEROLOGY HEPATOLOGY OR RHEUMATOLOGY)<br>Indexes=SCI-EXPANDED, SSCI, A&HCI, CPCI-S, CPCI-SSH, BKCI-S, BKCI-SSH, ESCI, CCR-EXPANDED, IC Timespan=1980-2017
#3	TOPIC: (model*) OR TOPIC: (regression) OR TOPIC: (odds ratio) OR TOPIC: (estimat*) OR TOPIC: ((projection* or projecting)) OR TOPIC: (Forecast*)<br>Indexes=SCI-EXPANDED, SSCI, A&HCI, CPCI-S, CPCI-SSH, BKCI-S, BKCI-SSH, ESCI, CCR-EXPANDED, IC Timespan=1980-2017
#2	TOPIC: (Epidemiology) OR TOPIC: (prevalence*) OR TOPIC: (burden*) OR TOPIC: (mortality) OR TOPIC: (incidence) OR TOPIC: (morbidity) OR TOPIC: (("disability-adjusted life years" or "disability adjusted life years" or DALY*)) OR TOPIC: ("years lived with disability") OR TOPIC: ("years life lost") OR TOPIC: ("Healthy life expectancy" or HALE) OR TOPIC: ("life expectancy") OR TOPIC: (cost*) OR TOPIC: ("cost of illness") OR TOPIC: ("health care cost$") OR TOPIC: ("drug cost$") OR TOPIC: ("hospital cost$") OR TOPIC: ("health expenditure$") OR TOPIC: (Hospitalization) OR TOPIC: ("Primary Health Care" or "Primary Care") OR TOPIC: ("Ambulatory Care") OR TOPIC: ("Emergency Service" or "Emergency visit") OR TOPIC: (absentee*)<br>Indexes=SCI-EXPANDED, SSCI, A&HCI, CPCI-S, CPCI-SSH, BKCI-S, BKCI-SSH, ESCI, CCR-EXPANDED, IC Timespan=1980-2017
#1	TOPIC: (Asthma*) OR TOPIC: ("Respiratory Sounds") OR TOPIC: (wheez*) OR TOPIC: ("Bronchial Spasm") OR TOPIC: (bronchospas*) OR TOPIC: ((bronch*

	NEAR/3 spasm*)) OR TOPIC: (bronchoconstrict*) OR TOPIC: ((antiasthma* or anti-asthma*)) OR TOPIC: (Bronchoconstriction) OR TOPIC: ((bronch* NEAR/3 constrict*)) OR TOPIC: ("Bronchial Hyperreactivity") OR TOPIC: ("Respiratory Hypersensitivity") OR TOPIC: ((bronchial* or respiratory or airway* or lung*) NEAR/3 (hypersensitiv* or hyperreactiv* or allerg* or insufficiency)) Indexes=SCI-EXPANDED, SSCI, A&HCI, CPCI-S, CPCI-SSH, BKCI-S, BKCI-SSH, ESCI, CCR-EXPANDED, IC Timespan=1980-2017

WHOLIS

1. asthma$ or "respiratory sounds" or wheez$ or "bronchial spasm" or bronchospas$ or bronchoconstrict$ or antiasthma$ or "bronchial hyperreactivity" or "respiratory hypersensitivity"
2. prevalence or burden or cost$ or incidence or mortality or "disability-adjusted life years" or DALY$ or "years lived with disability" or hospitalization or "primary care" or "ambulatory care" or emergency or absentee$
3. 1 AND 2

Table S1: Description of quality criteria in the checklist for assessing the quality of models for prevalence and burden of diseases

Checklist item	Description
Statement	There is a clear statement about the questions that the model aimed to answer
Structure	The model is defined with explicit structure/statement/formula
Appropriateness	The model is appropriate to deal with the nature, distribution and type of input data
Assumption	Necessary assumptions of the model are discussed in details
Model building	Performed model building (variable selection) and described the techniques/tools/criteria used
Model fitting	The process of model fitting is described in details
Model diagnosis	Performed model diagnostic and adequacy checking and methods are described in details
Testing Goodness of fit	Tested goodness of fit of the model and methods used for testing goodness of fit of the model are described in details
Missing data	Discussed how missing data were handled
Model validation	The model is validated adequately (including internal and external validation), and methods of validation are discussed in details
Sensitivity analysis	Effects of uncertainty were assessed by carrying out sensitivity analysis, and the methods are described in details
Model presentation	The final fitted model presented adequately including estimates of the parameters (intercepts and regression coefficients of the predictors) with standard errors and confidence interval
Reproducibility	Resources required to use/apply the model (method of model derivation, parameter estimation, drawing the inference, user manual, data/metadata/synthetic data, codes required to develop the model) are accessible (freely or under agreement) or publicly available so that other people can replicate/reproduce the model findings or can easily apply the model to similar other dataset.

Table S2: Summary of all included studies (summary of overall evidence)

Author	Context	Country	Outcome(s)	Burden measures	Case definition	Type of measurements	Period	Data type	Response variable	Data level	Data source	Model name
**Ding, 2017 [1]**	National	China	Burden	1. Quality of life;<br><br>2. Productivity impairment;<br><br>and<br><br>3. Healthcare utilisation	Two definitions were used to classify the severity of patients: (1) Global Initiative for Asthma (GINA) 2014 guidelines based on self-reported medication use and (2) self-reported asthma (physician-diagnosed) severity	Estimating mean outcomes of the burden measures for different levels of asthma severity in adults aged 18 or older	For the years 2010, 2012 and 2013	*Continuous data:* quality of life variables;<br><br>*Count data:* productivity impairment variables and healthcare utilisation variables	*Quality of life variables:* physical component summary score, mental component summary score, and health state utility score;<br><br>*Productivity impairment variables:* absenteeism (%), presenteeism (%), overall work impairment (%), and activity impairment (%);<br><br>*Healthcare utilisation variables:* number of healthcare provider visits, emergency room (ER) visits and hospitalisations	Individual level data	China National Health and Wellness Surveys (NHWS)	***Generalised linear models (GLM)***<br>[Normal distribution with identity link function for estimating quality of life outcomes; Negative binomial regression for estimating productivity impairment and healthcare utilisation]
**Bardal, 2017 [2]**	National	Canada	Both prevalence and burden	Healthcare service utilisation	Hospital admissions with asthma as the principal diagnosis based on ICD-9 code 493 or ICD-10 code J45, or physician visits for asthma as the principal diagnosis based on ICD-9 code 493, or having three or more asthma drug dispensing episodes	Estimating changes in the prevalence of asthma and healthcare service utilisation over time for patients aged 5 to 55 years	Period: 1996-2009	*Binary data*: presence of asthma;<br><br>*count data (rate)*: visit rates and hospital admission rate	Presence of asthma (yes/no);<br><br>Visit rates (number of visits per 100 patients per year) of family practitioner (FP), specialists and emergency department (ED);<br><br>Hospital admission rate (number of yearly hospital admissions for asthma per 100 admissions)	*Individual level data:* presence of asthma;<br><br>*Aggregate data:* yearly visit rates and hospital admission rate	British Columbia linked health services databases	***Logistic regression model*** for estimating prevalence of asthma (using presence of asthma as response variable);<br><br>***Poisson regression model*** for estimating rates of FP visit, specialists visit, ED visit and hospital admission
**Prietsch, 2012 [3]**	National	Brazil	Burden	Mortality	Asthma was recorded as the underlying cause of death according to ICD-9 code 493 or ICD-10 code J45	Estimating trend in asthma mortality among children up to 19 years old	Period: 1980-2007	Continuous data (rate)	Asthma mortality rate (number of asthma deaths/population, multiplied by 100,000)	Aggregate data (country-level)	Database of the Brazilian Unified Health System	***Linear regression model***
**Oganov, 1999 [4]**	National	Russia	Burden	Mortality	Asthma was recorded as the "underlying cause" of death according to ICD-9 code 493	Estimating annual changes in asthma death rates for the whole Russian population	Period: 1980-1989	Continuous data (rate)	Asthma mortality rate (number of deaths per 100,000 population per year)	Aggregate data (country level)	Statistical Department of the Ministry of Health of the Russian Federation	***Linear regression model***
**Pesut, 2011 [5]**	National	Serbia	Burden	Mortality	Asthma was recorded as the underlying cause of death according to ICD-9 code 493 and ICD-10 codes J45 and J46	Estimating trend of asthma mortality rate in the whole Serbian population as well as in the ≥5–34 year age group	Period: 1980-2009	Continuous data (rate)	Annual age specific mortality rates (number of asthma related deaths per 100,000 inhabitants) for all ages;<br><br>Annual age specific mortality rates for the ≥5–34 age group;<br><br>Annual gender specific mortality rates	Aggregate data (country level)	Death Registry of the Republic of Serbia Institute of Statistics	***Linear regression model*** for estimating the average absolute annual change in the asthma mortality rate;<br><br>***Log trasformed linear model*** for estimating the average relative annual change in the asthma mortality rate
**Chatkin, 2007 [6]**	National	Brazil	Burden	Mortality	Asthma was recorded as the underlying	Estimating trends in asthma	Period: 1981-2003	Continuous data (rate)	Asthma mortality rates	Aggregate data	Death certificates at the Division of	***Linear and quadratic regression models***

					cause of death according to ICD-9 code 493 and ICD-10 codes J45 and J46	mortality rate among individuals of ages 5 to 39 years					Health of the Department of Health, State of Rio Grande do Sul	
**Schleicher, 2000 [7]**	National	United States	Burden	Mortality	Asthma was listed as the underlying cause of death	Estimating annual changes in asthma mortality rates among youths (ages 5–17 years)	Period: 1981-1995	Count data (rate)	Yearly asthma mortality rates	Aggregate data	Death certificates provided by the California Departments of Vital Statistics; Population data from the U.S. Census Bureau	***Poisson regression model***
**Hassanzadeh, 2012 [8]**	National	Iran	Prevalence	-	Self-reported "ever asthma"	Estimating pooled asthma prevalence in school children (ages 11–16 years)	Period: 1997-2009	Binomial data (n=sample size, p = prevalence %)	study level prevalence and standard errors	Aggregate data (study level)	Electronic database search (Medline, Pub-med, ISI, Google Scholar, Scopus, and local databases such as, Scientific Information Database (SID), Iranmedex, and Irandoc)	***Meta- analysis: random effects model*** (DerSimonian-Laird method)
**Thanh, 2009 [9]**	National	Canada	Burden	Productivity losses	Self-reported-diagnosed asthma	Estimating number and cost of asthma-related productivity loss days in people of working age (18–64 years)	For the year 2005	Continuous data	Number of disability days per patient per year	Individual level data	2005 Canadian Community Health Survey	***Linear regression model***
**van Woensel, 2002 [10]**	National	Netherlands	Burden	Hospitalisation	Hospital admission for asthma recorded with ICD-9-CM code 493	Estimating trend in asthma hospitalisation for children aged 0–4 years	Period: 1991-1999	Count data	Total number of asthma hospitalisations	Aggregate data (country level)	Hospital discharge data the National Medical Registration (LMR Prismant, Utrecht, Netherlands)	***Linear regression model***
**Sullivan, 2011 [11]**	National	United States	Burden	Direct and indirect costs	The presence of asthma was determined by ICD-9 code 493. Patients who had some expenditure or encounter with the medical system within the year related to ICD-9 code 493 were classified as having asthma	Estimating direct and productivity-related costs of asthma in adults (≥18 years)	For the years 2003 and 2005	*Count data:* productivity (number of missed work days and number of days spent sick in bed per year), medical utilisation (office based visits, outpatient visits, emergency department visits, hospital discharges, and number of prescriptions) and comorbidity (number of comorbid conditions);<br><br>*Binary data:* productivity (activity	*Productivity:* number of missed work days, the number of days spent sick in bed per year, activity limitation, inability variables, employment status and annual wages;<br><br>*Medical utilisation*: total number of office based visits, outpatient visits, emergency department visits, hospital discharges, and number of prescriptions;<br><br>*Comorbidity* (number of comorbid conditions);<br><br>*Medical expenditure*	Individual level data	The 2003 and 2005 Medical Expenditure Panel Surveys	***Negative binomial regression and zero-inflated negative binomial regression models*** for estimating the number of missed work days, number of days spent sick in bed per year, office based visits, outpatient visits, emergency department visits, hospital discharges, number of prescriptions, and number of comorbid conditions;<br><br>***Logistic regression models*** for estimating activity limitation, inability variables, and employment status;<br><br>***Heckman selection models*** for estimating logarithmic transformation of annual wages and medical expenditure

								limitation, inability variables, employment status);<br><br>*Continuous data:* annual wages and medical expenditure				
**James, 2010 [12]**	National	Australia	Prevalence	-	Self-reported clinician-diagnosed asthma ever	Estimating current prevalence of asthma in adults (aged 18–79 years) and changes in prevalence over time	Over the years 1966, 1969, 1972, 1975, 1981, 1990 and 2005–2007	Binary data	Having asthma (yes/no)	Individual level data	Respiratory health surveys of adults in Busselton	***Logistic regression model*** for estimating changes in the prevalence of asthma;<br><br>***Generalised estimating equations (GEE)*** to accommodate repeated measures
**Malik, 2011 [13]**	National	United Kingdom	Prevalence	-	1. Self-reported lifetime (ever) asthma;<br><br>2. Self-reported wheeze in past 3 years	Estimating changes in the lifetime prevalence of asthma and trend in wheeze in school children aged 9–12 years	Over three survey years- 1999, 2004, 2009	*Continuous data:* Imputed values for lifetime asthma;<br><br>*Binary data*: wheeze	Imputed values for lifetime asthma;<br><br>Having wheeze in past 3 years (yes/no)	Individual level data	Primary Surveys	***Generalised linear model*** for estimating changes in the prevalence of lifetime asthma over the three surveys;<br><br>***Logistic regression model*** for estimating trend in wheeze
**Akinbami, 2016 [14]**	National	United States	Prevalence	-	Self-reported clinician-diagnosed and current asthma	Estimating trend in the prevalence of current asthma in children aged 0–7 years	Period: 2001-2013	Binary data	Having asthma (yes/no)	Individual level data	National Health Interview Survey data	***Logistic regression model***
**Kausel, 2013 [15]**	National	Chile	Prevalence	-	1. Self-reported asthma ever;<br><br>2. Self-reported current asthma symptoms (wheezing or whistling in the chest in the last 12 months)	Estimation: annual prevalence	For the year 2009	Binary data	Having asthma (yes/no)	Individual level data	Cross-sectional primary survey conducted in semi urban and rural sectors in the province of Valdivia	***Logistic regression model***
**Lincoln, 2006 [16]**	National	Australia	Burden	Hospital admission	Hospital admission for asthma recorded with ICD-9 code 493 and ICD-10 codes J44.8, J45 and J46	Estimating temporal trend in the hospital admission for asthma in children aged 1–14 years	Period: 1994-2000	Count data	Daily counts of hospital admissions for asthma	Aggregate data (country level)	Routinely collated admission records for New South Wales hospitals of the Inpatient Statistics Collection (ISC)	***Generalised additive model (GAM)*** with a log-link function and using penalised regression splines
**Nath, 2015 [17]**	National	United States	Burden	Emergency department (ED) visits	ED visits with a principal ICD-9-CM diagnosis code for (potentially preventable) asthma as specified in the Agency for Healthcare Research and Quality's asthma pediatric quality indicator	Estimating trend in the annual rate of ED visits for asthma per thousand children (aged 2–17 years)	Period: 2001-2010	Continuous data (rate)	Annual rate of ED visits for asthma per 1000 children	Aggregate data (country level)	National Hospital Ambulatory Medical Care Survey	***Weighted linear regression model***
**Rezvanfar, 2013 [18]**	National	Iran	Burden	Direct medical and non-medical costs	Asthma diagnosis by a specialist based on GINA 2009 guidelines	Estimating total annual country-level costs of childhood asthma and the mean	For the year 2012	Continuous data	Costs related to medications, physician visits, respiratory tests, travelling and accommodation	Individual level data	Population data on Children's Medical Center (a referral	***Cost assessment model***

						annual cost of asthma per child (under 16 years)					center of asthma treatment in Iran); medical records of patients; patients' data questionnaires including asthma control test questionnaire based on the GINA guidelines	
**Chouaid, 2004 [19]**	National	France	Burden	Direct costs	No information provided	Estimating mean annual cost of asthma per patient	For varying years	Count data	Number of stable patient, acute attack, hospitalisation and death due to asthma	Aggregate data (study level)	Published data in national literature	***First degree homogeneous Markov model***
**Barnett, 2011 [20]**	National	United States	Burden	Incremental direct medical costs and productivity losses	Self-reported medical events and prescription medications related to ICD-9-CM code of 493	Estimating individual and national level incremental direct medical costs and productivity losses due to asthma	Period: 2002-2007	*Continuous data:* total medical expenditure ; *Count data:* productivity losses	*Total medical expenditure* (sum of office-based medical provider expenditures, hospital outpatient expenditures, emergency department expenditures, hospital inpatient expenditures, and prescribed medicine expenditures); *Productivity losses:* number of school days lost and the number of work days lost per calendar year	Individual level data	The Medical Expenditure Panel Surveys 2002-2007	***Two-part models (first part using logistic regression and second part using gamma regression)*** for estimating the incremental direct costs; ***Negative binomial regression model*** for estimating the incremental productivity losses
**Trogdon, 2015 [21]**	National	United States	Both prevalence and burden	Medical and absenteeism costs	Self-reported treated asthma (medical events and prescription medications related to ICD-9-CM: 493; CCS: 128)	Estimation and projection of annual treated population prevalence of asthma, and medical and absenteeism costs of asthma	*Estimation period:* 2004-2008; *Projection period:* 2010-2020	*Binary data:* having treated for asthma (yes/no); *Continuous data:* per person annual medical cost; *Count data:* number of annual work day missed	*Treated population prevalence:* having treated for asthma (yes/no); *Medical cost:* per person annual medical cost (on a log scale); *Absenteeism:* number of annual work day missed	Individual level data	Multiple national data sets from various sources/payer populations (2008 US Census Bureau for all payers combined, fiscal year 2008 Medicaid Statistical Information Statistics for Medicaid, Kaiser Family Foundation 2008 Medicare Health and Prescription Drug Plan Tracker for Medicare, and 2008 Current Population Survey (CPS) for the privately insured); Treated population prevalence (2004–2008 Medical Expenditure Panel Survey)	***Survey weighted logistic regression model*** for estimating treated population prevalence; ***Generalised linear model with a gamma distribution and a log link function*** for estimating medical cost; ***Negative binomial regression models*** for estimating work day missed
**Tual, 2010 [22]**	National	France	Burden	Mortality	Asthma was recorded as the underlying cause of death (ICD-9 code 493 and ICD-10 codes J45 and J46)	Estimating annual average rate of change in asthma-related mortality	Period: 1980-2005	Count data (rate)	Age-standardised mortality rates ((on a natural logarithm scale)	Aggregate data (country metropolitan level)	CépiDc-Epidemiological Center on Medical Causes of Death, the French National Institute for Health and Medical Research Laboratory	***Linear regression model***
**Wang, 2005 [23]**	National	United States	Burden	Direct and indirect costs	Self-reported treated asthma (an event cost associated with an ICD-9 code 493)	Estimating annual costs of asthma per school-age	For the year 1996	*Continuous data:* excess all-cause	Excess all-cause medical costs; Excess school absence days	Individual level data	The 1996 Medical Expenditure Panel Survey	***Linear regression model***

						children (5–17 years)		medical costs;<br><br>*Count data:* excess school absence days					
**Sadatsafavi, 2010 [24]**	National	Canada	Burden	Direct healthcare costs	Narrow definition of physician visits: physician visits that were coded as asthma according to ICD-9 code 493.xx. Broad definition of physician visits: all physician visits for an asthma-related diagnosis;<br><br>Narrow definition of hospitalisation: hospitalisations in which asthma was coded as the 'most responsible' diagnosis (ie, ICD-9 493.xx). Broad definition of hospitalisation: all hospitalisations in which asthma was indicated among the discharge diagnoses	Estimating mean costs of asthma per patient (aged 5–55 years) in each study year and the trends over the study period	Period: 1996-2000	Continuous data	log(cost+1)	Individual level data	Administrative health care data from BC PharmaNet database and the BC Linked Health Database (BCLHD)	***Generalised linear mixed effect model***
**Zannetos, 2017 [25]**	National	Cyprus	Burden	Economic burden: direct and indirect costs	Diagnosis of asthma defined according to Expert panel report 3: guidelines for the diagnosis and management of asthma	Estimating costs of asthma per adult person per year	For the year 2015	Continuous data	Cost of asthma per patient	Individual level data	Primary data collected by sample survey	***Bootstrapped prevalence-based cost of illness model***
**Severien, 1998 [26]**	National	Germany	Burden	Hospitalisation	Diagnosis of bronchial asthma	Estimating the trend of asthma hospitalisation rates in children (aged 0–17 years) with acute asthma	Period: 1976-1995	Continuous data (rate)	Hospitalisation rates	Aggregate data (city level)	Clinical documentation of the Institute of Medical Biometry and Statistics of the Medical University of Lübeck	***Linear regression model***
**Sliwczynski, 2015 [27]**	National	Poland	Prevalence	-	Diagnosis with ICD-10 code: J45.X-bronchial asthma	Estimating the number of patients with asthma in each study year	Period: 2008-2012	Count data	Number of asthma patients	Individual level data	National Health Fund (NFZ) — reported by health care providers regarding the patients diagnosed with asthma	***Negative binomial regression model***
**Mukherjee, 2016 [28]**	National	United Kingdom	Burden	Healthcare costs	Varying case definitions depending on the datasets	Estimating health care costs of asthma per year in the UK and its member nations	Period: 2011-2012	Continuous data	Costs of healthcare utilisation (GP consultations, practice nurse consultation, out-of-hours care, community prescribing, ambulance, accident and emergency visits, inpatient episodes, intensive care unit (ICU) utilisation) and disability living allowance	Both individual level and aggregate data	27 datasets comprised of national health surveys for 2010–11, primary and secondary National Health Service (NHS) datasets, and routine administrative, health and social care linked national databases for 2011–12	***Economic model***

**Adeloye, 2013 [29]**	Regional	Africa	Prevalence	-	Self-reported asthma diagnosis, and/or its symptoms (wheeze at rest, wheeze on exercise, nocturnal wheeze, nocturnal cough, or severe wheeze)	Estimating asthma prevalence in Africa	For the years 1990, 2000, and 2010	Continuous data (rate)	Weighted mean prevalence	Aggregate data (study level)	Systematic literature search	***Non-linear exponential regression model***
**Mendez-Luck, 2007 [30]**	National	United States	Prevalence	-	Reported asthma symptom prevalence defined as ever having had a prior diagnosis of asthma and having asthma symptoms in the previous 12 months	Estimating prevalence of asthma symptoms for small areas	For the year 2001	Binary data	Asthma symptom prevalence (having one or more symptoms/no symptoms)	Both individual and aggregate data (zip code level)	2001 California Health Interview Survey, 2000 Census, and 2000–2002 Current Population Surveys	***Hierarchical logistic regressions model***
**CDC, 1998 [31]**	National	United States	Prevalence	-	Self-reported having asthma diagnosed by physician at some time in life and reported symptoms of asthma during the preceding 12 months)	Forecasting state-specific estimates of asthma prevalence	For the year 1998	Continuous data (rate)	Region specific asthma prevalence	Aggregate data (region-specific)	1995 National Health Interview Survey, U.S.	***Linear regression model***
**Soyiri, 2013 [32]**	National	United Kingdom	Burden	Hospital admission	Any hospital emergency admission with a primary diagnosis of asthma (according to ICD-10 code of J 45)	Forecasting number of daily asthma hospital admissions	Period: 2005-2006	Count data	Daily count of the asthma admissions	Aggregate data (hospital level)	Hospital Episode Statistics (HES 2008), National Health Service, England	***Quantile regression model***
**Suruki, 2017 [33]**	National	United Kingdom and United States	Burden	Asthma exacerbation	Patient recorded with asthma medical code (US: International Classification of Diseases, 9th Revision: 493.xx; UK: CPRD medical codes/Read codes)	Estimating mean annual asthma exacerbation rates per patient (aged ≥12 years)	Period: 2009-2011 in UK; 2010-2011 in US	Count data	Number of exacerbations per patient per year	Individual level data	US based Clinformatics™ DataMart Multiplan (IMPACT) database and UK-based Clinical Practice Research Datalink (CPRD).	***Negative binomial regression model***
**Soriano, 2017 [34]**	Global, regional, and national	195 countries and territories	Both prevalence and burden	Mortality	Reported diagnosis of asthma by a physician, with wheezing in the past 12 months (the relevant ICD-10 codes are J45 and J46. ICD-9 code is 493).	Estimating annual asthma prevalence and mortality	Period: 1990-2015	Continuous data (proportion and rate)	*Prevalence*: prevalence, incidence, and remission data points; *Mortality*: death rates	Aggregate data (national and sub-national level)	*Prevalence*: population surveys and literature data; *Mortality:* vital registration and surveillance data from the cause of death (COD) database	***DisMod-MR 2.1 (Bayesian meta-regression model)*** for estimating prevalence; ***Cause Of Death Ensemble modelling (CODEm)*** for estimating mortality *[CODEm: mixed effects models or spatiotemporal Gaussian process regression models]*
**Burney, 1990 [35]**	National	England	Prevalence	-	Reported diagnoses of asthma	Estimating annual trend (changes) in the prevalence of asthma in primary school children (aged 4–12 years)	Period: 1973-1986	Binary data	Whether the child had asthma in the past year (yes/no)	Individual level data	National study of health and growth in primary school children in England	***Logistic regression model***
**Lozano, 1997 [36]**	National	United States	Burden	Healthcare utilisation and costs	At least one diagnosis of asthma (based on ICD Code-9 of 493.XX) listed for an outpatient visit or hospitalisation (provider-diagnosed asthma)	Estimating marginal costs of healthcare utilisation per children (aged 1–17 years)	For the year 1992	Continuous data	Overall costs of asthma and costs of non-urgent outpatient visits, pharmacy fills, urgent care visits, and hospital days due to asthma	Individual level data	Group Health Cooperative of Puget Sound (GHC) information system in western Washington State	***Multivariate regression model with weighted least squares***

**Chinn, 2004 [37]**	National	14 countries	Prevalence	-	Self-reported asthma symptoms (wheeze, nocturnal tightness in chest, shortness of breath, coughing) and ‘‘diagnosed’’ asthma (asthma attacks in the last 12 months or current medication)	Estimating change in the prevalence of asthma per year of follow up in adults (aged 20–44 years)	Period: 10 years of follow up (1991-93 to 1998-2003)	Binary data	Having (yes/no) wheeze, wheeze with breathlessness, wheeze without a cold, woken with chest tightness, woken with shortness of breath, woken by attack of coughing, attack of asthma, current medication, diagnosed asthma (attack of asthma or current medication); Prevalence estimates by centre	*Individual level data:* Having asthma symptoms; *Aggregate data:* prevalence estimates by centre	European Community Respiratory Health Survey (ECRHS) I and II	***Generalised estimating equations (GEE)*** for binary outcomes; ***Meta- analysis: random effects model*** for combining prevalence estimates by centre
**Backman, 2017 [38]**	National	Sweden	Prevalence	-	Current asthma: a physician-diagnosed asthma in combination with at least one of (i) attacks of shortness of breath last 12 months, (ii) any wheeze last 12 months or (iii) current asthma medication use	Estimating prevalence trends (prevalence ratios) of current asthma among adults (aged 20–69 years)	Period: 1996-2016	Count data	Current asthma; allergic asthma; and non-allergic asthma	Individual level data	Primary survey data of Obstructive Lung Disease in Northern Sweden (OLIN) Studies	***Poisson regression model***
**Brozek, 2015 [39]**	National	Poland	Prevalence	-	Self-reported physician-diagnosed asthma ever	Estimating trend in the prevalence of asthma in children aged 7-10 years over a 21 year period	Period: 1993-2002-2007-2014	Binary data	Medical condition of asthma (yes/no)	Individual level data	Four repeated cross-sectional surveys performed in Chorzow, Poland in 1993, 2002, 2007, and 2014.	***Logistic regression model***
**Venn, 1998 [40]**	National	England	Prevalence	-	Self-reported currently active diagnosed asthma	Estimating current prevalence (1995) and change in prevalence (1995-1988) of asthma in British primary school children (aged 4–11 years)	For the year 1995; and change for the period of 1995-1988	Binary data	Having asthma (yes/no)	Individual level data	Cross-sectional data from parental questionnaire surveys in 1988 and 1995 in the Nottingham, England	***Logistic regression model***
**Kamble, 2009 [41]**	National	United States	Burden	Incremental direct expenditures	Asthma patients were indemnified as those with ICD-9-CM diagnosis codes 493.xx for asthma in 2004 or those who had a self-report of having asthma in 2004	Estimating annual mean incremental total expenditures and expenditures for various categories of resource use associated with asthma in children (age <18 years) and in adults (age $\geq$18 years).	For the year 2004	Continuous data	Seven response variables: total direct medical expenditures, prescribed medicines expenditures, ER visit expenditures, office-based medical provider visits expenditures, inpatient visits expenditures, outpatient visits expenditures, and other medical expenditures due to asthma	Individual level data	2004 Medical Expenditure Panel Survey	***Poisson regression models***
**Wijesinghe, 2009 [42]**	National	20 countries	Burden	Mortality	Deaths for asthma were certified according to the ICD codes in use during that time period	Estimating trends of asthma mortality rates in the 5–34 year age group	Period: 1960-2005	Continuous data (rate)	Number of asthma deaths per 100,000 persons	Aggregate data (country level)	Centers for Disease Control and Prevention Wide-ranging Online Data for Epidemiologic Research (orWONDER); Eurostat Web site; and personal correspondence.	***LOESS (locally weighted regression) model***
**To, 2013 [43]**	National	Canada	Both prevalence and burden	Incidence	Individuals who had at least two asthma outpatient claims in	Projecting the incidence and prevalence for the	For the years 2012 and 2022	Continuous data	Annual incidence rate per 1000 population	Aggregate data	The Ontario Asthma Surveillance Information System	***Double exponential smoothing model***

					two consecutive years or at least one hospitalisation for asthma	years 2012 and 2022		(proportion and rate)		(province level)	(OASIS): linked health administrative databases	
**Verlato, 2003 [44]**	National	Italy	Prevalence	-	1. Self-report of an attack of asthma in the last 12 months;<br><br>2. Asthma-like symptoms (i. wheezing, ii. wheezing with breathlessness, iii. wheezing without a cold, iv. Chest tightness, and v. shortness of breath) in the last 12 months;<br><br>3. currently taking any medicines for asthma	Estimating temporal variations in the prevalence (change in prevalence) of asthma and asthma-like symptoms in adults aged 20–44 years	Between two survey periods (1991-93 and 1998-2000)	Binary data	Having an attack of asthma (yes/no);<br><br>Asthma-like symptoms (yes/no): 1. wheezing, 2. wheezing with breathlessness, 3. wheezing without a cold, 4. chest tightness, and 5. shortness of breath;<br><br>Currently taking any medicines for asthma (yes/no)	Individual level data	Primary survey conducted for Italian Study on Asthma in Young Adults (ISAYA)	***Logistic regression model***
**Brogger, 2003 [45]**	National	Norway	Prevalence	-	Self-reported asthma diagnosis: ever treated by a doctor or admitted to hospital for asthma;<br><br>Wheezing (whistling sound) ever in the chest;<br><br>Attacks of shortness of breath	Estimating change in prevalence of asthma symptoms and diagnosis in adults aged 15–70 years	Between two survey periods (1972 and 1998-99)	Binary data	Asthma diagnosis (yes/no);<br><br>Wheezing (yes/no);<br><br>Attacks of breathlessness (yes/no)	Individual level data	Two serial large-scale cross-sectional general population asthma survey were performed in Oslo in 1972 and 1998–1999	***Logistic regression model with regression splines***
**Alvarez-Alvarez, 2016 [46]**	Regional	World regions (Europe, Latin America and Africa)	Prevalence	-	Wheezing and/or recurrent wheezing (no definition for wheezing was specified; recurrent wheezing was defined as three or more episodes of wheezing)	Estimating prevalence of wheezing and recurrent wheezing in infants aged up to two years	Period: 2001-2016	Continuous data (rate)	Prevalence of asthma extracted from the included studies	Aggregate data (study level)	Literature search	***Meta-analysis: random effects Bayesian model***
**Ungar, 1998 [47]**	National	Canada	Burden	Direct and indirect costs	Patients who had a prescription for a bronchial inhaler medication and who reported experiencing shortness of breath, wheeze or recurrent cough in the past	Estimating annual cost of asthma per adult	Period: 1995-1996	Continuous data	Total costs of asthma (direct costs: costs of visits to general/family practitioners, specialists, emergency rooms, hospital admissions, laboratory tests, prescription medications, dispensing fees, devices and out-of-pocket expenses; indirect costs: costs of absences, restricted activity days, travel and waiting time)	Individual level data	Registrants in the Pharmacy Medication Monitoring Program (PMMP) Bronchial Inhalers project	***Log transformed linear regression model***
**Dai, 2017 [48]**	National	United States	Prevalence	-	Self-reported current asthma (ever-told asthma and still have asthma)	Predicting asthma prevalence in adults across geographic regions	For the year 2015	Continuous data (rate)	Asthma prevalence rate	Aggregate data (geographic region level)	The Social Media Monitoring (SMM) data from Twitter; the 2014 Behavioural Risk Factor Surveillance System (BRFSS); the 2014 American	***General linear predictive model***

											Community Survey (ACS)	
**Ram, 2015 [49]**	National	United States	Burden	Emergency department (ED) visits	ED visits for asthma as a primary diagnosis (ICD9 code 493.00 to 493.99)	Predicting the number of asthma-related emergency department (ED) visits per day in a specific area	For the year 2013	Count data	Asthma ED visit counts	Aggregate data (specific geographic area level)	Children's Medical Centre (CMC) of Dallas, Twitter data, Google search interest data, and environmental sensor data from EPA databases	***Machine learning (decision tree and artificial neural networks) based prediction model***
**Rosychuk, 2015 [50]**	National	Canada	Burden	Emergency department (ED) visits	Nosologists-recorded diagnosis of asthma-either the first or second diagnosis field containing the diagnostic codes 493.x (ICD-9-CM) or J45.x (ICD-10-CA)	Estimating temporal trends in the ED visits rate for asthma during a 12 year period;<br><br>Forecasting ED visits rate for asthma for the 24 months following the study period	Period: 2000-2011	Continuous data (rate)	Monthly asthma ED visit rates per 100,000 population	Aggregate data (province level)	National Ambulatory Care Reporting System (NACRS)	***Seasonal autoregressive integrated moving average (SARIMA) models***
**Miller, 2016 [51]**	National	United States	Both prevalence and burden	Health-care costs associated with asthma	Individuals having asthma who was included in the medical condition files using ICD-9 code and Clinical Classification Codes (CCC: 128)	Estimating prevalence of asthma and health-care costs associated with asthma per year in children aged 0–18 years	Period: 2005-2012	Binary data: having asthma (yes/no);<br><br>Continuous data: annual medical expenditure per children	Having asthma (yes/no);<br><br>Annual medical expenditure per children	Individual level data	2005–2012 Medical Expenditure Panel Surveys (MEPS)	***Logistic regression model*** for estimating prevalence;<br><br>***Two-part models (first part using logistic regression and second part using generalised linear model with a log link and gamma distribution)*** for estimating costs
**Lopez-Silvarrey-Varela, 2011 [52]**	National	Spain	Prevalence	-	ISAAC definition: self-reported asthma ever and recent asthma symptoms (wheezing in the last 12 months; and wheezing with exercise in the last 12 months)	Estimating prevalence of asthma and asthma symptoms in children (aged 6–7 years) and adolescent (aged 13–14 years)	Period: 2006-2007	Binary data	Asthma ever (yes/no);<br><br>Recent wheezing (yes/no);<br><br>Recent wheezing with exercise (yes/no)	Individual level data	Population survey conducted following ISAAC methodology in the school setting in Galicia, Spain	**Logistic regression model**
**Mohammadbeigi, 2011 [53]**	National	Iran	Prevalence	-	Self-reported "ever had asthma" based on ISAAC questionnaire	Estimating prevalence of asthma over the period in elementary school children aged 6–12 years	Period: 1999-2007	Continuous data (rate)	Prevalence of asthma extracted from the included studies	Aggregate data (study level)	Literature search	***Meta-analysis: random effects model***
**Varmaghani, 2016 [54]**	National	Iran	Prevalence	-	1. Asthma ever;<br><br>2. Current asthma;<br><br>3. Exercise-induced asthma;<br><br>4. Physician-diagnosed asthma	Estimating prevalence of asthma for each definition	Period: 1990-2015	Continuous data (rate)	Prevalence of asthma for each definition extracted from the included studies	Aggregate data (study level)	Literature search	***Meta-analysis: random effects model***
**Solis Soto, 2014 [55]**	National	Bolivia	Prevalence	-	Self-reported asthma symptoms (wheeze ever, wheeze-12 months prevalence) and severe asthma symptoms based on ISAAC standardized questionnaire ;	Estimating urban and rural prevalence of asthma symptoms for each definition in fifth grade school-aged children	For the year 2011	Binary data	Asthma symptoms (yes/no)	Individual level data	Population-based sample survey using ISAAC written and video questionnaire, conducted in the Oropeza province in 2011	***Generalised linear mixed effect model***

					Self-reported wheezing, exercise-induced wheeze and dyspnoea at rest based on ISAAC video questionnaire							
**So, 1990 [56]**	National	Hong Kong	Burden	Mortality	Asthma death recorded with ICD-9 code 490 and 493	Estimating trend of asthma deaths in the 5-34 years age group	Period: 1976-1985	Continuous data (rate)	Logarithm of the standardized mortality ratios (SMRs)	Aggregate data (yearly)	Government Census and Statistics Department of Hong Kong	***Log transformed linear regression model***
**Sullivan, 2017 [57]**	National	United States	Burden	School absence and productivity outcomes	Presence of current asthma in conjunction with ICD-9 diagnosis code 493 “Asthma” (ever diagnosed with asthma and still have asthma)	Estimating annual missed school days and missed work days of parents/caregivers of school-aged children (aged 6–11 years) and adolescents (aged 12–17 years)	Period: 2007-2013	Count data	Number of missed school days due to asthma;<br><br>Number of work days missed to care for others having asthma	Individual level data	2007–2013 Medical Expenditure Panel Survey (MEPS)	***Negative binomial regression*** for estimating missed school days;<br><br>***Two-part models (first part using probit model and second part using negative binomial regression)*** for estimating missed work days
**Linneberg, 2001 [58]**	National	Denmark	Prevalence	-	Diagnoses of allergic asthma for ‘asthma related to pollen exposure’, ‘asthma related to dander exposure’, and ‘asthma related to mite exposure’. Furthermore, 12-month period prevalence were defined by combining these symptoms with ‘shortness of breath when at rest at any time in last 12 months’	Estimating changes in the prevalence of allergic asthma symptoms in adults aged 15-41 years	Period: 1989-1998	Binary data	Asthma related to pollen exposure (yes/no);<br><br>Asthma related to dander exposure (yes/no);<br><br>Asthma related to mite exposure (yes/no);<br><br>12-month period prevalence of allergic asthma symptoms (yes/no)	Individual level data	Two cross-sectional surveys, conducted in 1989 and 1998 in the western part of Copenhagen County (Denmark)	***Logistic regression model***
**Tzeng, 1998 [59]**	National	Taiwan	Burden	Mortality	No information provided	Estimating asthma mortality rate per 1,000,000 male aged 25-59 years	For the year 1991	Count data	Number of asthma deaths	Aggregate data (area level)	Health and vital statistics 1991, Republic of China	**Conditional Autoregressive (CAR) model;**<br><br>**Exchangeable (EX) Model – Poisson-Gamma Model**
**Nurmagambetov, 2017 [60]**	National	United States	Both prevalence and burden	Medical and absenteeism cost	Medically treated asthma recorded using ICD-9-CM code 493 and CCS code 128	Estimating prevalence of asthma, per person and total medical costs, per capita and total number of workdays lost, and absenteeism costs for all ages and for children aged 0-7 years;<br><br>Projecting medical costs from 2015 to 2020 for the entire population and separately for children	Estimation for the year 2012;<br><br>Projection for the years 2015-2020	*Binary data:* prevalence;<br><br>*Continuous data:* medical expenditure;<br><br>*Count data:* number of workdays and schooldays lost	Having asthma (yes/no);<br><br>Per person total annual medical expenditure;<br><br>Annual per capita number of workdays and schooldays lost	Individual level data	Multiple data sources, including 2008-2012 Medical Expenditure Panel Survey (for the main analysis), U.S. Census Bureau, Kaiser Family Foundation, Medical Statistical Information System, and Current Population Survey	***Logistic regression model*** for estimating and projecting prevalence;<br><br>***Two-part models (first part: logistic regression and second part: generalised linear model with a gamma distribution and a log link)*** for estimating and projecting medical expenditure;<br><br>***Negative binomial model*** for estimating and projecting number of workdays and schooldays lost
**Entezari, 2009 [61]**	National	Iran	Prevalence	-	Patient reported outcomes of asthma symptoms measured	Estimating prevalence of asthma symptoms	Period: 1998-2003	Continuous data (rate)	Prevalence of asthma symptoms extracted from the included studies	Aggregate data (study level)	Literature search	***Meta-analysis: random effects model***

					using ISAAC protocol	in children aged <18 years						
**Frank 2005 [62]**	National	England	Prevalence	-	Reported adult asthma symptoms (wheeze, being woken by cough, being woken by tightness in the chest, being woken by shortness of breath (all in the previous year));<br><br>Reported childhood asthma was defined by three or more of the five questions on: wheeze, night cough, more than three course of antibiotics (all in the past year), history of hayfever/eczema, and family history of asthma	Estimation: changes in the prevalence of asthma symptoms in adults and asthma in children aged <16 years	Period: 1993–2001	Binary data	Having (yes/no) asthma symptoms in adults;<br><br>Having asthma in children (yes/no)	Individual level data	A series of postal questionnaire surveys conducted in 1993, 1995, 1999, and 2001 by Wythenshawe Community Asthma Project (WYCAP)	***Generalised Estimating Equations (GEE)***
**Kolokotroni, 2011 [63]**	National	Cyprus	Prevalence	-	Defined based on ISAAC questionnaire:<br><br>1. Ever wheeze;<br><br>2. Current wheeze;<br><br>4. Asthma diagnosis ever;<br><br>5. Severity of asthma	Estimating temporal changes in the prevalence of asthma in children aged 7–8 years	Period: 2000-2008	Binary data	Ever wheeze (yes/no);<br><br>Current wheeze (yes/no);<br><br>Asthma diagnosis ever (yes/no);<br><br>Severity of asthma (yes/no)	Individual level data	Two cross sectional surveys conducted using ISAAC questionnaire during 1999-2000 and 2007-08 on the children enrolled in the second grade of public primary schools (i.e. aged 7-8 years) in Nicosia and Limassol in Cyprus	***Logistic regression model***
**Ng, 1999 [64]**	National	Singapore	Burden	Mortality	Asthma death recorded with ICD-8: 493, ICD-9: 493, ICD-10: J45, J46	Estimating temporal trends in the asthma mortality rate	Period: 1976-1995	Count data	Annual numbers of deaths from asthma	Aggregate data (country level)	Government departments in Singapore responsible for keeping vital statistics: the Department of Statistics from 1976 to 1988 and the National Registration Department from 1989 to 1995	***Poisson regression model***
**Luis Lopez-Campos, 2008 [65]**	National	Spain	Burden	Mortality	Death from asthma coded as 493 in ICD-8 and ICD-9 between 1975 and 1998, and J45–46 in ICD-10 for the period 1999–2005	Estimating temporal trends in the asthma mortality rate	Period: 1975-2005	Continuous data (rate)	Standardised asthma mortality rates	Aggregate data (country level)	Official publications of the Instituto de Estad´ıstica de Andaluc´ıa	***Joinpoint regression model***
**Tavakoli, 2017 [66]**	National	Canada	Burden	Direct costs	Validated case definition of physician-diagnosed asthma: patients with one asthma-related hospitalisation or two or more physician	Estimating the excess costs of asthma per year and their trend in the population aged 5–55 years	Period: 2002-2011	Continuous data	Excess direct medical costs due to asthma	Individual level data	Population-based provincial administrative health databases of British Columbia	***Generalised Estimating Equations (GEE)***

					visits with the main diagnosis of asthma or three or more dispensation records for asthma-related medications (in different dates) in a rolling 12-month window were considered asthmatic							
**Chew, 1998 [67]**	National	Singapore	Burden	Hospital admissions and Accident and Emergency room (A&E) visits	Hospital admissions and A&E visits for asthma coded as ICD 9: 493	Estimating trends and seasonal variation in hospital admissions and A&E visits for childhood asthma (children < 12 years)	Period: 1990-1996	Continuous data (rate)	Rates of A&E visits and hospital admissions per million population due to astma	Aggregate data (hospital level)	National University Hospital and Singapore General Hospital	***Box-Jenkins regression-ARIMA model***
**Zhang, 2013 [68]**	National	United States	Prevalence	-	Self-reported current asthma (clinician diagnosed asthma ever and still have asthma)	Estimating state-specific current asthma prevalence in adults by 2-year periods and temporal changes in asthma prevalence	Period: 2000-2009	Binary data	Current asthma status (yes/no)	Individual level data	Behavioural Risk Factor Surveillance System (BRFSS) data from all 50 states and the District of Columbia	***Logistic regression model***
**Grauden z, 2017 [69]**	National	Brazil	Burden	Mortality	Asthma listed as the underlying cause of death using ICD-9 code 493 and ICD-10 codes J45-46	Estimating trends in asthma mortality for two age groups: 0–4 years and 5-34 years	Period: 1980-2014	Continuous data (rate)	Standardized asthma mortality rates	Aggregate data (country level)	Mortality Database maintained by the Information Technology Department of the Brazilian Unified Health Care System	***Polynomial regression model***
**Vicendes e, 2014 [70]**	National	Australia	Burden	Hospital re-admissions	Primary diagnosis of asthma as indicated by ICD-9 codes (493) and ICD-10 codes (J45 or J46)	Estimating trends in asthma hospital re-admission rates among children and adolescents aged 2-18 years	Period: 1997-2009	Binary data	Have two or more admissions due to asthma (yes/no);<br><br>Experience a repeat admission within 28 days due to asthma (yes/no);<br><br>Experience a repeat admission within one year due to asthma (yes/no)	Individual level data	Victorian Admitted Episodes Data set (VAED), Department of Health Victoria Australia	***Logistic regression model***
**Bedouch, 2012 [71]**	National	Canada	Burden	Direct medical costs	Patients were considered to have asthma if, during a 12-month rolling window, they had at least one asthma-related hospitalisation ((ICD-9: 493.x and ICD-10: J45, J46); two or more physician visits with diagnostic code of asthma (ICD-9:493.x); or filled four or more prescriptions for asthma-related medications	Estimating direct asthma-related medical costs, and trends in annual total and per-patient costs (sum of hospitalisations, physician visits, and medications costs) in the population aged 5–55 years	Period: 2002-2007	Continuous data (rate)	Annual total and per-patient asthma related costs	Individual level data	Linked administrative health data of British Columbia: The Discharge Abstracts Database (DAD); Medical Services Plan (MSP); The Vital Statistics database; Demographics and Registration databases; provincial PharmaNET system	***Generalised Linear Model with a gamma distribution and a logarithmic link function***

**Chua, 2011 [72]**	National	Australia, Hong Kong and Singapore	Burden	Hospitalisation	Principal diagnosis of asthma coded according ICD9-CM: 493 or ICD-10-AM: J45-46	Estimating time trends in yearly asthma hospitalisation counts for each country in children aged 0–14 years	Period: 1994-2008	Count data	Number of age-specific asthma hospitalisation each year	Aggregate data (country level)	Australian National Hospital Morbidity Database and the Australian Bureau of Statistics; the Hospital Authority – governing body for public hospitals in Hong Kong; and the Ministry of Health in Singapore	***Negative binomial regression model***
**Jang, 2013 [73]**	National	United States	Burden	Cost and functional outcomes	Lifetime diagnosis using 2 methods: (1) respondents who had ever been diagnosed with asthma and (2) respondents having an ICD-9-CM code of 493.x in any of the medical condition files	Estimating level and trends of medical expenditure, health status and functional outcomes in adults (≥18 years), adolescents (6–17 years), and children (0–5 years)	Period: 2000-2009	Continuous data	Health status (physical and mental component summary scores);<br><br>Functional outcomes (workdays and schooldays lost);<br><br>Medical expenditures	Individual level data	2000-2009 Medical Expenditure Panel Survey Household Component	***Linear regressions model*** for estimating health status;<br><br>***Generalised linear model with a gamma distribution and a log link function*** for estimating functional outcomes;<br><br>***Two-part models (first part: logit model and second part: generalised linear models with a log link and a gamma distribution)*** for estimating medical expenditures
**Antunes, 2012 [74]**	National	Brazil	Burden	Hospitalisation	Hospitalisations due to asthma were classified according to the corresponding ICD-10 code for asthma	Estimating trend of annual hospitalisation rates	Period: 1998-2009	Continuous data (rate)	Annual asthma hospitalisation rates	Aggregate data (country level)	DATASUS website: Hospital Information System (SIH), Brazil	***Linear regression model***
**Ebmeier, 2017 [75]**	National	46 countries	Burden	Mortality	Death those coded in ICD-9 as 493 and in ICD-10 as J45 or as J46	Estimating trend in the age-standardised country-specific asthma mortality rates in the 5–34 year age group	Period: 1993-2012	Continuous data (rate)	Age-standardised asthma mortality rate	Aggregate data (country level)	WHO Mortality Database	***LOESS (locally weighted scatter plot smoother) model***
**Manfreda, 1993 [76]**	National	Canada	Prevalence	-	Primary diagnosis of asthma using physician services as either inpatients or outpatients in a year (a single diagnosis is recorded on each physician claim and is coded according to the ICD-9: 493)	Estimating trend (average annual increase) of physician-diagnosed asthma prevalence	Period: 1980-1990	Continuous data (rate)	Annual prevalence of asthma	Aggregate data (country level)	Routinely collected data of the provincial Health Insurance Plan (physicians' claims) administered by the Manitoba Health Services Commission (MHSC)	***Linear regression model***
**de Marco, 2012 [77]**	National	Italy	Prevalence	-	Self-reported current asthma (an attack of asthma in the last 12 months and currently (in the last 12 months) taking any medicine for asthma) and asthma-like symptoms (wheezing or whistling; tightness in the chest; shortness of breath at any time in the last 12 months)	Estimating trends in the prevalence of current asthma and asthma-like symptoms in adults aged 20–44 years	Period: 1991-2010	*Binary data:* Current asthma and asthma-like symptoms;<br><br>*Count data:* median of the adjusted prevalence rates of asthma and asthma-like symptoms	Current asthma (present versus absent);<br><br>Asthma-like symptoms (present versus absent);<br><br>Median of the adjusted prevalence rates of asthma and asthma-like symptoms	*Individual level data*: current asthma and asthma-like symptoms;<br><br>*Aggregate data (yearly)*: Median of the adjusted prevalence rates of	European Community Respiratory Health Survey (ECRHS; 1991-93), the Italian Study on Asthma in Young Adults (ISAYA; 1998-2000) and the Gene Environment Interactions in Respiratory Diseases (GEIRD; 2007-2010)	***Logistic regression model*** for estimating prevalence rate;<br><br>***Poisson regression models*** for estimating trend in prevalence

										asthma and asthma-like symptoms		
**Kim, 2014 [78]**	National	Sweden	Prevalence	-	1. Self-reported physician diagnosed asthma (ever had asthma or asthmatic bronchitis and it was diagnosed by a physician);<br><br>2. Self-reported wheeze in 12 months;<br><br>3. Self-reported asthma symptoms (either had asthmatic symptoms or had both wheeze and breathlessness with tightness in the chest in 12 months);<br><br>4. Self-reported asthma medication during the last 12 months (both having physician diagnosed asthma and used any asthma medication during the last 12 months)	Estimating trends in prevalence of physician diagnosed asthma, asthma symptoms, wheeze and asthma medication in adolescents attending 9th grade at school (aged 15 years)	Period: 2000-2008	Binary data	Physician diagnosed asthma (present versus absent);<br><br>Asthma symptoms (present versus absent);<br><br>Wheeze (present versus absent);<br><br>Asthma medication (present versus absent)	Individual level data	Two cross-sectional surveys called "Allergy 2000 (All000)", and "Youth in Vastra Gotaland County," conducted in 2000 and 2008 respectively in Vastra Gotaland County, Sweden	***Logistic regression model***
**Gonzalez -Barcala, 2012 [79]**	National	Spain	Burden	Mortality	Cases which had asthma as a cause of death	Estimating trends in asthma deaths	Period: 1993-2007	Count data	Asthma death counts for each calendar year, age and gender	Aggregate data (country level)	Official death certificate from the Deaths Register of the Department of Public Health of the Regional Government of Galicia	***Poisson regression model***
**Huang, 2015 [80]**	National	China	Prevalence	-	Self-reported wheeze ever, wheeze in 12 months and doctor diagnosed asthma defined following ISAAC questionnaire	Estimating current prevalence of asthma and wheeze and time-trend in the prevalence of asthma in preschool children aged 3–7 years	Period: 1990-2011	Continuous data (rate)	Prevalence of asthma and wheezing	Aggregate data (country level)	CCHH (China, Children, Homes, Health) cross-sectional study conducted in 2011-12 in Shanghai; and systematic review of literature	***Exponential regression model***
**Uphoff, 2017 [81]**	National	Eight European countries	Prevalence	-	Parent-reported wheeze and parent-reported doctor-diagnosed asthma ever	Estimating prevalence rates of childhood (at age 4 years) asthma, wheeze and wheeze with diagnosed asthma	Period varies depending on cohort	Binary data	Asthma (present versus absent);<br><br>Wheeze (present versus absent);<br><br>Wheeze with asthma (present versus absent)	Individual level data	10 MeDALL (Mechanisms of the Development of ALLergy) cohorts in eight countries in Europe	***Logistic regression model***
**Newson, 1998 [82]**	National	England	Burden	Hospital admission	Acute asthma attacks	Forecasting daily asthma admissions counts for two age groups (0–14 years and ≥15 years)	Period: 1987-1994	Count data	Daily asthma admissions	Aggregate data (regional health authority level)	Hospital Episodes System (HES); UK Meteorological Office; Pollen Research Unit, Worcester, UK	***Log-linear autoregression model***

**Arathimos, 2017 [83]**	National	United Kingdom	Prevalence	-	In the Avon Longitudinal Study of Parents and Children (ALSPAC): reports of children's 1) asthma and wheeze symptoms in the past 12 months and 2) doctor's diagnosis of asthma ever;<br><br>In the Millennium Cohort Study (MCS): reports of 1) asthma ever, 2) wheeze ever and 3) wheeze in the last 12 months	Estimating sex discordance in the prevalence of childhood asthma and wheeze in different ages	ALSPAC study: cohort (born 1991-92) data from age 0-18 years;<br><br>MCS study: cohort (born 2000-02) data from age 3-10 years	Binary data	Asthma in the last 12 months (present versus absent);<br><br>Wheeze in the last 12 months (present versus absent);<br><br>Ever asthma (present versus absent);<br><br>Ever wheeze (present versus absent)	Individual level data	Two UK birth cohorts; the Avon Longitudinal Study of Parents and Children (ALSPAC) and the Millennium Cohort Study (MCS)	***Logistic regression model using restricted cubic splines***
**Ito, 2002 [84]**	National	Japan	Burden	Mortality	Asthma death coded as 241 in ICD 6th–8th revisions, as 493 ICD 9th revision, and as J45-J46 in ICD 10th revision	Estimating trends (age and cohort effects) in asthma mortality rates in the 5–34 years of age	Period: 1950-1997	Continuous data (rate)	Age-specific asthma mortality rates	Aggregate data (country level)	Certified asthma deaths obtained from The National Vital Statistics, published annually by the Ministry of Health and Welfare	***Multiplicative models for rates (Breslow and Day method)***
**Hoogendoorn, 2006 [85]**	National	Netherlands	Both prevalence and burden	Direct cost (healthcare utilisation and medication costs)	Health-care utilisation recorded with ICD-code 493 and asthma medication recorded with diagnosis code R96	Estimating and projecting prevalence of asthma, health-care utilisation and medication costs	Estimation for the year 2000;<br><br>Projection for the period of 2000-2025	Continuous data	Asthma-specific epidemiological data: prevalence, incidence, remission and mortality;<br><br>Asthma-specific cost of health-care utilisation and medication	Aggregate data (country level: gender, age and smoking specific)	Representative national registries and surveys including the Continuous Morbidity registration from Nijmegen, the Transition project from Amsterdam and the Registration network for Huisartsen Limburg from Maastricht, Periodic examination of living situation' by the Central Bureau of Statistics, Database National Information Network GPs in Utrecht, National Medical Registration in Utrecht	***RIVM Chronic disease model***
**Suijkerbuijk, 2013 [86]**	National	Netherlands	Both prevalence and burden	Healthcare costs	No information provided	Estimating and projecting prevalence and the societal costs of asthma	Estimation for the year 2007;<br><br>Projection for the for the period of 2007-2032	Continuous data	Asthma specific incidence, prevalence, remission and mortality;<br><br>Healthcare costs and productivity losess	Aggregate data (country level and age-specific)	Different GP registries including website of the National Compass (www.nationaalkompas.nl); nationwide registration records and representative patient panels including patient panel NPCG, PIAMA study, NIVEL study respiratory allergy, National Medical, Information project Registration, Dutch Transplantation Foundation; The National Information	***Computer simulation model***

											Network for General Practice Care (LINH) GIP-database on medicines and medical aids from the Health Care Insurance Board, 'Working with chronic lung disease' study	
**El-Sharif, 2003 [87]**	National	Palestine	Prevalence	-	Report of physician-diagnosed asthma and asthma symptoms (wheezing ever, 12-month wheezing, nocturnal cough and exercise-related wheezing)	Estimating prevalence of asthma and asthma symptoms in schoolchildren aged 5–8 year and 12–15 years	Period: 2000-2001	Binary data	Physician-diagnosed asthma (present versus absent); Asthma symptoms (present versus absent)	Individual level data	ISAAC phase III study: survey conducted using ISAAC phase III questionnaires and protocols in Ramallah and North Gaza in Palestine	***Logistic regression model***
**Bauman, 1993 [88]**	National	Australia	Prevalence	-	Diagnosed asthma and reported recent (12 month) and lifetime wheeze	Estimating prevalence and trend of diagnosed asthma and asthma symptoms in children aged 5–12 years	Period: 1969-1992	Continuous data (rate)	Prevalence of asthma and wheezing	Aggregate data (country level)	Seventeen population based studies: published epidemiological surveys of childhood asthma	***Linear regression model***
**Chatkin, 1999 [89]**	National	Brazil	Burden	Mortality	Asthma was reported to be the underlying cause of death using the codes ICD-8 (1970 to 1978) and ICD-9: 493 (1979-1992)	Estimating trends in mortality from asthma in children and young adults aged 5–39 years	Period: 1970-1992	Continuous data (rate)	Asthma mortality rates	Aggregate data (country level)	Division of Health Information of the Department of Health of the State of Rio Grande do Sul	***Linear and log-linear regression model***
**Vos, 2017 [90]**	Global, regional, and national	195 countries	Both prevalence and burden	Incidence	Doctor's diagnosis of asthma and wheezing in the past year; the relevant ICD-10 codes are J45 and J46, and ICD-9 code is 493	Estimating prevalence and incidence of asthma by age, sex, year, and location	Period: 1990–2016	Continuous data (rate)	Incidence, prevalence, remission, and death rates	Aggregate data (national and sub-national level)	Systematic review of the literature on asthma, various survey data, claims data from the United States from 2000, 2010, and 2012	***DisMod-MR 2.1*** (Bayesian meta-regression model)
**Naghavi, 2017 [91]**	Global, regional, and national	195 countrie s and territor ies	Burden	Mortality	Deaths assigned to ICD-10 codes J45-J46.9, and ICD-9 codes 493-493.9	Estimating asthma mortality by age, sex, year, and location	Period: 1980–2016	Continuous data (rate)	Death rates	Aggregate data (national and sub-national level)	Vital registration and surveillance data from the cause of death (COD) database	***Cause of Death Ensemble modelling (CODEm)*** (mixed effects linear models and spatial-temporal Gaussian process regression models)
**Vos, 2015 [92]**	Global, regional, and national	188 countries	Prevalence	-	Asymptomatic asthma (asthma, currently without symptoms); asthma, controlled (has wheezing and cough once a month, which does not cause difficulty with daily activities); asthma, partially controlled (has wheezing and cough once a week, which causes some difficulty with daily activities); asthma, uncontrolled (has wheezing, cough and shortness of breath	Estimating prevalence of asthma by age, sex, year, and location	Period: 1990–2013	Continuous data (rate)	Prevalence, incidence, excess mortality and remission	Aggregate data (national and sub-national level)	Systematic review of the literature on asthma and various survey data	***DisMod-MR 2.0*** (Bayesian meta-regression model)

					more than twice a week, which causes difficulty with daily activities and sometimes wakes the person at night). The relevant ICD-10 codes J45-J46.9, Z82.5, and ICD-9 codes 493-493.92, V17.5							
**Naghavi, 2015 [93]**	Global, regional, and national	188 countries	Burden	Mortality	Deaths assigned to ICD-10 codes J45-J46.9, and ICD-9 codes 493-493.92	Estimating asthma mortality by age, sex, year, and location	Period: 1990–2013	Continuous data (rate)	Death rates	Aggregate data (country level)	Vital registration and verbal autopsy	**Cause of death ensemble modelling (CODEm)** (non-linear mixed effects model, spatial-temporal regression and Gaussian process regression model)
**Lozano, 2012 [94]**	Global and regional	21 world regions (187 countries)	Burden	Mortality	Deaths assigned to ICD-10 codes J45-J46, and ICD-9 codes 493	Estimating asthma mortality by age, sex, year, and region	Period: 1990–2010	Continuous data (rate)	death rate; cause fraction	Aggregate data (country level)	Vital registration, verbal autopsy and surveillance	***Cause of death ensemble modelling (CODEm)*** (the best performing model or ensemble is selected from the mixed effects linear models of the log of the death rate, mixed effects linear models of the logit of the cause fraction, spatial-temporal Gaussian process regression (ST-GPR) models of the log of the death rate, and ST-GPR of the logit of the cause fraction)
**Vos, 2012 [95]**	Global and regional	21 world regions (187 countries)	Prevalence	-	No information provided	Estimating prevalence of asthma by age, sex, year, and region	Period: 1990–2010	Continuous data (rate)	Incidence, prevalence, remission, and excess mortality	Aggregate data (country level)	Systematic review of the literature on asthma and various survey data	***DisMod-MR*** (Bayesian meta-regression model)
**Rutten-van Mölken, 1999 [96]**	National	Netherlands	Both prevalence and burden	Direct cost (healthcare utilisation and medication costs)	In inpatient care data, asthma was defined with code ICD-9: 493;<br><br>In prevalence and incidence data, asthma was defined with code 0860 (asthma/asthmatic bronchitis) from the E-list	Projecting prevalence and direct costs (healthcare utilisation and medication costs) of asthma	For the year 2010	Continuous data	Age- and gender-specific incidence, prevalence, recovery and mortality for asthma	Aggregate data (country level: age and gender specific)	Representative national registries and large, representative surveys including Health Interview Survey conducted by Statistics Netherlands during 1992-1994, National Medical Registration and nursing-home registration provided by SIG Health Care Information. Information on the incidence and prevalence of asthma was available from a continuous regional registration among Dutch GPs. Recovery and mortality data were computed using published recovery rates and case fatality rates.	***Epidemiological model based on a dynamic multi-state lifetable***

**World Health Organiza tion, 2008 [97]**	Global and regional	Six WHO regions, and 14 sub-regions	Both prevalence and burden	Mortality, incidence and YLD	Disease categorised with the ICD-10 codes J45-J46	Estimating asthma prevalence, incidence, YLD and mortality by age, sex and region and projecting trends in asthma mortality	Estimation: for the year 2004;<br><br>Projection: for the period 2004-2030	Continuous data (rate)	Age-and-sex-specific incidence, prevalence, remission and death rate	Aggregate data (country leve)	Population-based epidemiological estimates based on studies, vital registration system, sample registration systems, disease registers, and surveillance systems	***DisMod II*** for estimating prevalence, incidence and YLD;<br><br>***Cause-of-death modelling (CodMod)*** for estimating mortality;<br><br>***Linear regression model*** for projecting mortality
**Mathers, 2003 [98]**	Global and regional	Six WHO regions, and 14 and 17 sub-regions	Both prevalence and burden	Mortality, incidence and YLD	For prevalence: reported wheeze in the last 12 months plus a positive airway hyperresponsiveness test (current bronchial hyperresponsiveness, defined as a 20% fall in FEV1 with a provoking concentration of histamine at 8 mg/ml or less);<br><br>For mortality: deaths assigned to ICD-9 code 493 and ICD-10 codes J45-J46	Estimating asthma prevalence, incidence YLD and mortality by age, sex and region	For the year 2002	Continuous data (rate)	Age-and-sex-specific incidence, prevalence, remission and death rate	Aggregate data (country leve)	Population-based epidemiological estimates based on studies, vital registration system, sample registration systems, disease registers, and surveillance systems	***DisMod II*** for estimating prevalence, incidence and YLD;<br><br>***Cause-of-death modelling (CodMod)*** for estimating mortality
**Mathers, 2006 [99]**	Global and regional	106 countries	Burden	Mortality	Deaths assigned to ICD-10 codes J45-J46, and ICD-9 codes 493	Projecting asthma mortality	Period: 2002–2030	Continuous data (rate)	Age-and-sex-specific death rate in the logarithmic scale	Aggregate data (country level)	Death registration data	***Linear regression model***
**Mathers, 2006 [100]**	Global and regional	Six WHO regions, and 17 sub-regions	Both prevalence and burden	Mortality, incidence and YLD	For prevalence: reported wheeze in the last 12 months plus a positive airway hyperresponsiveness test (current bronchial hyperresponsiveness, defined as a 20% fall in FEV1 with a provoking concentration of histamine at 8 mg/ml or less);<br><br>For mortality: deaths assigned to ICD-9 code 493 and ICD-10 codes J45-J46	Estimating asthma prevalence, incidence YLD and mortality by age, sex and region	For the year 2001	Continuous data (rate)	Age-and-sex-specific Incidence, prevalence, remission and death rate	Aggregate data (country level)	Population-based epidemiological estimates based on studies, vital registration system, sample registration systems, disease registers, and surveillance systems	***DisMod II*** for estimating prevalence, incidence and YLD;<br><br>***Cause-of-death modelling (CodMod)*** for estimating mortality
**Mathers, 2002 [101]**	Global and regional	Six WHO regions, and 14 sub-regions	Both prevalence and burden	Mortality, incidence and YLD	For prevalence: reported wheeze in the last 12 months plus a positive airway hyperresponsiveness test (current bronchial hyperresponsiveness, defined as a 20% fall in FEV1 with a provoking concentration of histamine at 8 mg/ml or less);	Estimating asthma prevalence, incidence YLD and mortality by age, sex and region	For the year 2000	Continuous data (rate)	Age-and-sex-specific Incidence, prevalence, remission death rate	Aggregate data (country-level)	Disease registers, population surveys, epidemiological studies, and health facility data	***DisMod*** for estimating prevalence, incidence and YLD;<br><br>***Cause-of-death modelling (CodMod)*** for estimating mortality

					For mortality: deaths assigned to ICD-9 code 493 and ICD-10 codes J45-J46							
**Jarvis, 2018 [102]**	National	13 European countries and Australia	Prevalence	-	1. Self-reported symptoms (wheeze, wheeze with breathlessness, wheeze in the absence of a cold, woken with chest tightness, woken with shortness of breath, woken by attack of coughing);<br><br>2. Attacks of asthma in the last 12 months;<br><br>3. Current use of asthma medication;<br><br>4. Diagnosed asthma	Estimating changes in the prevalence of symptoms, asthma attack, current asthma medication and diagnosed asthma between different survey periods in adults	Period: three surveys over 20 years (1990–1992, 2000–2002 and 2008–2013)	*Binary data:* symptoms, asthma attack, current asthma medication and diagnosed asthma;<br><br>*Continuous data (rate):* asthma prevalence by centre	Symptoms (presence/absence);<br><br>Asthma attack (presence/absence);<br><br>Current asthma medication (presence/absence);<br><br>Diagnosed asthma (presence/absence);<br><br>Asthma prevalence by centre	*Individual level data:* symptoms, asthma attack, current asthma medication and diagnosed asthma;<br><br>*Aggregate data:* asthma prevalence by centre	The European Community Respiratory Health Survey I, II and III	***Generalised estimating equations (GEE)*** for estimating prevalence of symptoms, asthma attack, current asthma medication and diagnosed asthma;<br><br>***Meta- analysis: random effects model*** for combining prevalence estimates by centre
**Cohen, 2015 [103]**	National	Israel	Both prevalence and burden	Hospitalisation and death rates	1. Lifetime asthma (diagnosed);<br><br>2. Asthma severity: i) asthma in remission, ii) intermittent asthma, iii) mild persistent asthma, iv) moderate-to-severe persistent asthma;<br><br>3. Asthma-related hospital admissions listed as the ICD-9 code 493.xx;<br><br>4. Asthma death where the cause of death on the death certificate was: 'asthma', 'asthma exacerbation' or 'status asthmaticus' (based on ICD-10)	Estimating trends in the annual asthma prevalence, severity, hospitalisation and death rates in 17-year-old boys	Period: 1999-2008	Continuous data (rate)	Annual asthma prevalence;<br><br>Annual asthma severity: asthma in remission, intermittent asthma, mild persistent and moderate-to-severe asthma;<br><br>Annual asthma hospitalisation;<br><br>Annual asthma death rates	Aggregate data (country level)	Medical records of a cohort of 17-year-old males who underwent a comprehensive medical evaluation for eligibility for national service between 1999 and 2008; National Hospital Discharges Database of the Ministry of Health Information Department; Central Bureau of Statistics of Israel (Registry of Causes of Death)	***Linear regression model***
**Nurmagambetov, 2018 [104]**	National	United States	Burden	Incremental medical costs and absenteeism cost	Treated asthma defined as the presence of at least one medical or pharmaceutical encounter or claim associated with asthma	Estimating incremental medical costs of asthma and absenteeism cost (missed work and school days)	Period: 2008-2013	*Continuous data:* expenditure;<br><br>*Binary data:* expenditure (positive/no);<br><br>*Count data:* absenteeism	Annual per-person total healthcare expenditure (or medical cost);<br><br>Annual per-person expenditures for each of the following services: office visits, hospital outpatient visits, ER visits, hospital admissions and prescription medications;<br><br>Having a positive healthcare expenditure (yes/no);<br><br>Missed workdays and school days	Individual level data	2008–2013 household component of the Medical Expenditure Panel Surveys	***Two-part models (first part using logistic regression and second part using GLM with a log link and gamma distribution)*** for estimating expenditures;<br><br>***Negative binomial regression model*** for estimating absenteeism

**Morris, 1994 [105]**	National	United States	Burden	Hospital admissions	Primary hospital discharge diagnosis recorded using ICD-9CM code 493	Estimating annual hospital admissions rate for asthma among the elderly	Period: 1984-1989	Continuous data (rate)	Sex-specific rates and combined rates of hospital admission due to asthma	Aggregate data (county level)	Hospital admissions data for Medicare patients maintained by the Health Care Financing Administration (HCFA)	***Linear regression model***
**Luyt, 1993 [106]**	National	England	Prevalence	-	Report of doctor-diagnosed asthma;<br><br>Report of attacks of wheezing ever;<br><br>Report of attacks of wheezing during the past 12 months;<br><br>Report of recurrent cough without cold	Estimating cumulative prevalence of wheeze and doctor diagnosed asthma and the point prevalence of recurrent cough and wheeze in children aged ≤5 years	For the year 1990	Binary data	Number of children with each case definition and total number of children in each age-sex group	Aggregate data (age and sex specific)	Questionnaire survey of population based random sample of children registered on regional authority's child health index for immunisation in Leicestershire	***Logistic regression model***
**Goren, 1997 [107]**	National	Israel	Prevalence	-	Report of asthma and wheezing with shortness of breath based on American Thoracic Society-National Heart and Lung Institute (ATS-NHLI) self-administered Questionnaire	Estimating temporal trend and changes in the prevalence of asthma and wheezing with shortness of breath	Period: 1980-1989	Binary data	Asthma/wheeze outcomes (present versus absent)	Individual level data	Four cross-sectional data sets gathered among eighth-grade schoolchildren (aged 13–14 yrs) as part of health monitoring system operated in the vicinity of a new power plant in Israel	***Logistic regression model***
**Aguinaga, 1999 [108]**	National	Spain	Prevalence	-	Asthma ever and recent wheeze (last 12 months) based on ISAAC written and video questionnaire	Estimating prevalence rates of asthma ever and recent wheeze in 13–14-year-old children	Period: 1993-1995	Count data	Asthma count	Aggregate data (region and sex specific)	Nine Spanish populations (children aged 13-14 years in Almería, Barcelona, Bilbao, Bahía de Cádiz, Cartagena, Castellón, Pamplona, Valencia and Valladolid) were interviewed using the asthma written and video questionnaires of the ISAAC protocol	***Poisson regression model***

References

1 Ding B, DiBonaventura M, Karlsson N, Ling X. A cross-sectional assessment of the prevalence and burden of mild asthma in urban China using the 2010, 2012, and 2013 China National Health and Wellness Surveys. J Asthma. 2017;54:632-43.

2 Bardal S, Smith A, Luo HA, Zhang T, Groeneweg G, Jimenez Mendez R, et al. Asthma in British Columbia: Are we finally breathing easier? A population-based study of the burden of disease over 14 years. J Asthma. 2017;54:308-17.

3 Prietsch SOM, Zhang L, Catharino AR, Vauchinski L, Rodrigues FE. Asthma mortality among Brazilian children up to 19 years old between 1980 and 2007. J Pediatr (Rio J). 2012;88:384-8.

4 Oganov RG, Maslennikova GY. Asthma mortality in Russia between 1980 and 1989. Eur Respir J. 1999;13:287-9.

5 Pesut DP, Bulajic MV, Nagomi-Obradovic LM, Grgurevic AD, Gledovic ZB, Ponomarev DR, et al. Asthma mortality in Serbia: A 30-year analysis. Respir Med. 2011;105:S50-S3.

6 Chatkin G, Chatkin JM, Fritscher CC, Cavalet-Blanco D, Bittencourt HR, Sears MR. Asthma mortality in southern Brazil: Is there a changing trend? J Asthma. 2007;44:133-6.

7 Schleicher NC, Koziol JA, Christiansen SC. Asthma mortality rates among California youths. J Asthma. 2000;37:259-65.

8 Hassanzadeh J, Mohammadbeigi A, Mousavizadeh A, Akbari M. Asthma prevalence in Iranian guidance school children, a descriptive meta-analysis. J Res Med Sci. 2012;17:293-7.

9 Thanh NX, Ohinmaa A, Yan C. Asthma-related productivity losses in Alberta, Canada. J Asthma Allergy. 2009;2:43-8.

10 van Woensel JB, van Aalderen WM, Kneyber MC, Heijnen ML, Kimpen JL. Bronchiolitis hospitalisations in the Netherlands from 1991 to 1999. Arch Dis Child. 2002;86:370-1.

11 Sullivan PW, Ghushchyan VH, Slejko JF, Belozeroff V, Globe DR, Lin SL. The burden of adult asthma in the United States: Evidence from the medical expenditure panel survey. J Allergy Clin Immunol. 2011;127:363-9.e3.

12 James AL, Knuiman MW, Divitini ML, Hui J, Hunter M, Palmer LJ, et al. Changes in the prevalence of asthma in adults since 1966: the Busselton health study. Eur Respir J. 2010;35:273-8.

13 Malik G, Tagiyeva N, Aucott L, McNeill G, Turner SW. Changing trends in asthma in 9-12 year olds between 1964 and 2009. Arch Dis Child. 2011;96:227-31.

14 Akinbami LJ, Simon AE, Rossen LM. Changing Trends in Asthma Prevalence Among Children. Pediatrics. 2016;137:e20152354.

15 Kausel L, Boneberger A, Calvo M, Radon K. Childhood Asthma and Allergies in Urban, Semiurban, and Rural Residential Sectors in Chile. Sci World J. 2013;2013:937935.

16 Lincoln D, Morgan G, Sheppeard V, Jalaludin B, Corbett S, Beard J. Childhood asthma and return to school in Sydney, Australia. Public Health. 2006;120:854-62.

17 Nath JB, Hsia RY. Children's emergency department use for asthma, 2001-2010. Acad Pediatr. 2015;15:225-30.

18 Rezvanfar MA, Kebriaeezadeh A, Moein M, Nikfar S, Gharibnaseri Z, Abdollahi-Asl A. Cost analysis of childhood asthma in Iran: A cost evaluation based on referral center data for asthma and allergies. J Res Pharm Pract. 2013;2:162-8.

19 Chouaid C, Vergnenegre A, Vandewalle V, Liebaert F, Khelifa A, Galipon, et al. [The costs of asthma in France: an economic analysis by a Markov model]. Rev Mal Respir. 2004;21:493-9.French.
20 Barnett SB, Nurmagambetov TA. Costs of asthma in the United States: 2002-2007. J Allergy Clin Immunol. 2011;127:145-52.
21 Trogdon JG, Murphy LB, Khavjou OA, Li R, Maylahn CM, Tangka FK, et al. Costs of Chronic Diseases at the State Level: The Chronic Disease Cost Calculator. Prev Chronic Dis. 2015;12:E140.
22 Tual S, Godard P, Bousquet J, Annesi-Maesano I. The decrease in asthma-related mortality in France. Rev Mal Respir. 2010;27:e1-5.
23 Wang LY, Zhong Y, Wheeler L. Direct and indirect costs of asthma in school-age children. Prev Chronic Dis. 2005;2:A11.
24 Sadatsafavi M, Lynd L, Marra C, Carleton B, Tan WC, Sullivan S, et al. Direct health care costs associated with asthma in British Columbia. Can Respir J. 2010;17:74-80.
25 Zannetos S, Zachariadou T, Zachariades A, Georgiou A, Talias MA. The economic burden of adult asthma in Cyprus; a prevalence-based cost of illness study. BMC Public Health. 2017;17:262.
26 Severien C, Friedrich HJ. Entwicklung der Hospitalisationsraten für das Asthma bronchiale im Kindesalter. Monschr Kinderheilkd. 1998;146:951-6.Germany.
27 Sliwczynski A, Brzozowska M, Iltchew P, Czeleko T, Kucharczyk A, Jedrzejczyk T, et al. Epidemiology of asthma in Poland in urban and rural areas, based on provided health care services. Pneumonol Alergol Pol. 2015;83:178-87.
28 Mukherjee M, Stoddart A, Gupta RP, Nwaru BI, Farr A, Heaven M, et al. The epidemiology, healthcare and societal burden and costs of asthma in the UK and its member nations: analyses of standalone and linked national databases. BMC Med. 2016;14:113.
29 Adeloye D, Chan KY, Rudan I, Campbell H. An estimate of asthma prevalence in Africa: a systematic analysis. Croat Med J. 2013;54:519-31.
30 Mendez-Luck CA, Yu H, Meng YY, Jhawar M, Wallace SP. Estimating health conditions for small areas: asthma symptom prevalence for state legislative districts. Health Serv Res. 2007;42:2389-409.
31 Centers for Disease Control and Prevention (CDC). Forecasted state-specific estimates of self-reported asthma prevalence--United States, 1998. MMWR Morb Mortal Wkly Rep. 1998;47:1022-5.
32 Soyiri IN, Reidpath DD, Sarran C. Forecasting peak asthma admissions in London: an application of quantile regression models. Int J Biometeorol. 2013;57:569-78.
33 Suruki RY, Daugherty JB, Boudiaf N, Albers FC. The frequency of asthma exacerbations and healthcare utilization in patients with asthma from the UK and USA. BMC Pulm Med. 2017;17:74.
34 Soriano JB, Abajobir AA, Abate KH, Abera SF, Agrawal A, Ahmed MB, et al. Global, regional, and national deaths, prevalence, disability-adjusted life years, and years lived with disability for chronic obstructive pulmonary disease and asthma, 1990-2015: a systematic analysis for the Global Burden of Disease Study 2015. Lancet Respir Med. 2017;5:691-706.
35 Burney PGJ, Chinn S, Rona RJ. Has the prevalence of asthma increased in children? Evidence from the national study of health and growth 1973-86. BMJ. 1990;300:1306-10.
36 Lozano P, Fishman P, VonKorff M, Hecht J. Health care utilization and cost among children with asthma who were enrolled in a health maintenance organization. Pediatrics. 1997;99:757-64.

37 Chinn S, Jarvis D, Burney P, Luczynska C, Ackermann-Liebrich U, Anto JM, et al. Increase in diagnosed asthma but not in symptoms in the European Community Respiratory Health Survey. Thorax. 2004;59:646-51.
38 Backman H, Raisanen P, Hedman L, Stridsman C, Andersson M, Lindberg A, et al. Increased prevalence of allergic asthma from 1996 to 2006 and further to 2016-results from three population surveys. Clin Exp Allergy. 2017;47:1426-35.
39 Brozek G, Lawson J, Szumilas D, Zejda J. Increasing prevalence of asthma, respiratory symptoms, and allergic diseases: Four repeated surveys from 1993-2014. Respir Med. 2015;109:982-90.
40 Venn A, Lewis S, Cooper M, Hill J, Britton J. Increasing prevalence of wheeze and asthma in Nottingham primary schoolchildren 1988-1995. Eur Respir J. 1998;11:1324-8.
41 Kamble S, Bharmal M. Incremental direct expenditure of treating asthma in the United States. J Asthma. 2009;46:73-80.
42 Wijesinghe M, Weatherall M, Perrin K, Crane J, Beasley R. International Trends in Asthma Mortality Rates in the 5-to 34-Year Age Group A Call for Closer Surveillance. Chest. 2009;135:1045-9.
43 To T, Stanojevic S, Feldman R, Moineddin R, Atenafu EG, Guan J, et al. Is asthma a vanishing disease? A study to forecast the burden of asthma in 2022. BMC Public Health. 2013;13:254.
44 Verlato G, Corsico A, Villani S, Cerveri I, Migliore E, Accordini S, et al. Is the prevalence of adult asthma and allergic rhinitis still increasing? Results of an Italian study. J Allergy Clin Immunol. 2003;111:1232-8.
45 Brogger J, Bakke P, Eide GE, Johansen B, Andersen A, Gulsvik A. Long-term changes in adult asthma prevalence. Eur Respir J. 2003;21:468-72.
46 Alvarez-Alvarez I, Niu H, Guillen-Grima F, Aguinaga-Ontoso I. Meta-analysis of prevalence of wheezing and recurrent wheezing in infants. Allergol Immunopathol (Madr). 2018;46:210-7.
47 Ungar WJ, Coyte PC, Chapman KR, MacKeigan L. The patient level cost of asthma in adults in south central Ontario. Pharmacy Medication Monitoring Program Advisory Board. Can Respir J. 1998;5:463-71.
48 Dai H, Lee BR, Hao J. Predicting Asthma Prevalence by Linking Social Media Data and Traditional Surveys. Ann Am Acad Polit Soc Sci. 2017;669:75-92.
49 Ram S, Zhang W, Williams M, Pengetnze Y. Predicting asthma-related emergency department visits using big data. IEEE J Biomed Health Inform. 2015;19:1216-23.
50 Rosychuk RJ, Youngson E, Rowe BH. Presentations to Alberta emergency departments for asthma: a time series analysis. Acad Emerg Med. 2015;22:942-9.
51 Miller GF, Coffield E, Leroy Z, Wallin R. Prevalence and Costs of Five Chronic Conditions in Children. J Sch Nurs. 2016;32:357-64.
52 Lopez-Silvarrey-Varela A, Pertega-Diaz S, Rueda-Esteban S, Sanchez-Lastres JM, San-Jose-Gonzalez MA, Sampedro-Campos M, et al. Prevalence and geographic variations in asthma symptoms in children and adolescents in Galicia (Spain). Arch Bronconeumol. 2011;47:274-82.
53 Mohammadbeigi A, Hassanzadeh J, Mousavizadeh A. Prevalence of asthma in elementary school age children in Iran--a systematic review and meta analysis study. Pak J Biol Sci. 2011;14:887-93.
54 Varmaghani M, Farzadfar F, Sharifi F, Rashidian A, Moin M, Moradi-Lakeh M, et al. Prevalence of Asthma, COPD, and Chronic Bronchitis in Iran: A Systematic Review and Meta-analysis. Iran J Allergy Asthma Immunol. 2016;15:93-104.

55 Solis Soto MT, Patino A, Nowak D, Radon K. Prevalence of asthma, rhinitis and eczema symptoms in rural and urban school-aged children from Oropeza Province - Bolivia: a cross-sectional study. BMC Pulm Med. 2014;14:40.

56 So SY, Ng MMT, Ip MSM, Lam WK. Rising asthma mortality in young males in Hong Kong, 1976-85. Respir Med. 1990;84:457-61.

57 Sullivan P, Ghushchyan VG, Navaratnam P, Friedman HS, Kavati A, Ortiz B, et al. School absence and productivity outcomes associated with childhood asthma in the USA. J Asthma. 2018;55:161-8.

58 Linneberg A, Nielsen NH, Madsen F, Frolund L, Dirksen A, Jorgensen T. Secular trends of allergic asthma in Danish adults. The Copenhagen Allergy Study. Respir Med. 2001;95:258-64.

59 Tzeng JY, Hsiao CK, Chen CJ. Spatial model selection using Bayes factor and ratio of variabilities for asthma mortality data. Chin J Public Health. 1998;17:158-69.

60 Nurmagambetov T, Khavjou O, Murphy L, Orenstein D. State-level medical and absenteeism cost of asthma in the United States. J Asthma. 2017;54:357-70.

61 Entezari A, Mehrabi Y, Varesvazirian M, Pourpak Z, Moin M. A systematic review of recent asthma symptom surveys in Iranian children. Chron Respir Dis. 2009;6:109-14.

62 Frank PI, Wicks PD, Hazell ML, Linehan MF, Hirsch S, Hannaford PC, et al. Temporal change in the prevalence of respiratory symptoms and obstructive airways disease 1993-2001. Br J Gen Pract. 2005;55:596-602.

63 Kolokotroni O, Middleton N, Nicolaou N, Pipis S, Priftis KN, Milton DK, et al. Temporal changes in the prevalence of childhood asthma and allergies in urban and rural areas of Cyprus: results from two cross sectional studies. BMC Public Health. 2011;11:858.

64 Ng TP, Tan WC. Temporal trends and ethnic variations in asthma mortality in Singapore, 1976-1995. Thorax. 1999;54:990-4.

65 Luis Lopez-Campos J, Cayuela A, Rodriguez-Dominguez S, Vigil E. Temporal trends in asthma mortality over 30 years. J Asthma. 2008;45:611-4.

66 Tavakoli H, FitzGerald JM, Chen W, Lynd L, Kendzerska T, Aaron S, et al. Ten-year trends in direct costs of asthma: a population-based study. Allergy. 2017;72:291-9.

67 Chew FT, Goh DY, Ooi BC, Lee BW. Time trends and seasonal variation in acute childhood asthma in tropical Singapore. Respir Med. 1998;92:345-50.

68 Zhang X, Morrison-Carpenter T, Holt JB, Callahan DB. Trends in adult current asthma prevalence and contributing risk factors in the United States by state: 2000-2009. BMC Public Health. 2013;13:1156.

69 Graudenz GS, Carneiro DP, Vieira RP. Trends in asthma mortality in the 0- to 4-year and 5- to 34-year age groups in Brazil. J Bras Pneumol. 2017;43:24-31.

70 Vicendese D, Abramson MJ, Dharmage SC, Tang ML, Allen KJ, Erbas B. Trends in asthma readmissions among children and adolescents over time by age, gender and season. J Asthma. 2014;51:1055-60.

71 Bedouch P, Marra CA, FitzGerald JM, Lynd LD, Sadatsafavi M. Trends in Asthma-Related Direct Medical Costs from 2002 to 2007 in British Columbia, Canada: A Population Based-Cohort Study. PLoS ONE. 2012;7:e50949.

72 Chua KL, Ma S, Prescott S, Ho MHK, Ng DK, Lee BW. Trends in childhood asthma hospitalisation in three Asia Pacific countries. J Paediatr Child Health. 2011;47:723-7.

73 Jang J, Gary Chan KC, Huang H, Sullivan SD. Trends in cost and outcomes among adult and pediatric patients with asthma: 2000-2009. Ann Allergy Asthma Immunol. 2013;111:516-22.
74 Antunes FP, Costa MDN, Paim JS, Vieira-da-Silva LM, Santos C, Cruz AA, et al. Trends in hospitalizations for respiratory diseases in Salvador, Bahia State, Brazil, 1998-2009. Cad Saude Publica. 2012;28:869-77.
75 Ebmeier S, Thayabaran D, Braithwaite I, Benamara C, Weatherall M, Beasley R. Trends in international asthma mortality: Analysis of data from the WHO Mortality Database from 46 countries (1993-2012). The Lancet. 2017;390:935-45.
76 Manfreda J, Becker AB, Wang PZ, Roos LL, Anthonisen NR. Trends in physician-diagnosed asthma prevalence in Manitoba between 1980 and 1990. Chest. 1993;103:151-7.
77 de Marco R, Cappa V, Accordini S, Rava M, Antonicelli L, Bortolami O, et al. Trends in the prevalence of asthma and allergic rhinitis in Italy between 1991 and 2010. Eur Respir J. 2012;39:883-92.
78 Kim JL, Brisman J, Aberg MA, Forslund HB, Winkvist A, Toren K. Trends in the prevalence of asthma, rhinitis, and eczema in 15 year old adolescents over an 8 year period. Respir Med. 2014;108:701-8.
79 Gonzalez-Barcala FJ, Aboal J, Carreira JM, Rodriguez-Alvarez MX, Puga A, Sanjose E, et al. Trends of asthma mortality in Galicia from 1993 to 2007. J Asthma. 2012;49:1016-20.
80 Huang C, Liu W, Hu Y, Zou Z, Zhao Z, Shen L, et al. Updated prevalences of asthma, allergy, and airway symptoms, and a systematic review of trends over time for childhood asthma in shanghai, China. PLoS ONE. 2015;10:e0121577.
81 Uphoff EP, Bird PK, Anto JM, Basterrechea M, von Berg A, Bergstrom A, et al. Variations in the prevalence of childhood asthma and wheeze in MeDALL cohorts in Europe. ERJ Open Res. 2017;3:00150-2016.
82 Newson R, Strachan D, Archibald E, Emberlin J, Hardaker P, Collier C. Acute asthma epidemics, weather and pollen in England, 1987-1994. Eur Respir J. 1998;11:694-701.
83 Arathimos R, Granell R, Henderson J, Relton CL, Tilling K. Sex discordance in asthma and wheeze prevalence in two longitudinal cohorts. PLoS ONE. 2017;12:e0176293.
84 Ito Y, Tamakoshi A, Wakai K, Takagi K, Yamaki K, Ohno Y. Trends in asthma mortality in Japan. J Asthma. 2002;39:633-9.
85 Hoogendoorn M, Feenstra TL, Rutten-Van Molken MPMH. [Projections of future resource use and the costs of asthma and COPD in the Netherlands]. Ned Tijdschr Geneeskd. 2006;150:1243-50.Dutch.
86 Suijkerbuijk AWM, De Wit GA, Wijga AH, Heijmans MJWM, Hoogendoorn M, Rutten-Van Molken MPMH, et al. [Societal costs of asthma, COPD and respiratory allergy]. Ned Tijdschr Geneeskd. 2013;157:A6562.Dutch.
87 El-Sharif NA, Nemery B, Barghuthy F, Mortaja S, Qasrawi R, Abdeen Z. Geographical variations of asthma and asthma symptoms among schoolchildren aged 5 to 8 years and 12 to 15 years in Palestine: the International Study of Asthma and Allergies in Childhood (ISAAC). Ann Allergy Asthma Immunol. 2003;90:63-71.
88 Bauman A. Has the prevalence of asthma symptoms increased in Australian children? J Paediatr Child Health. 1993;29:424-8.
89 Chatkin JM, Barreto SM, Fonseca NA, Gutierrez CA, Sears MR. Trends in asthma mortality in young people in southern Brazil. Ann Allergy Asthma Immunol. 1999;82:287-92.

90 Vos T, Abajobir AA, Abate KH, Abbafati C, Abbas KM, Abd-Allah F, et al. Global, regional, and national incidence, prevalence, and years lived with disability for 328 diseases and injuries for 195 countries, 1990-2016: a systematic analysis for the Global Burden of Disease Study 2016. Lancet. 2017;390:1211-59.
91 Naghavi M, Abajobir AA, Abbafati C, Abbas KM, Abd-Allah F, Abera SF, et al. Global, regional, and national age-sex specific mortality for 264 causes of death, 1980-2016: a systematic analysis for the Global Burden of Disease Study 2016. Lancet. 2017;390:1151-210.
92 Vos T, Barber RM, Bell B, Bertozzi-Villa A, Biryukov S, Bolliger I, et al. Global, regional, and national incidence, prevalence, and years lived with disability for 301 acute and chronic diseases and injuries in 188 countries, 1990-2013: a systematic analysis for the Global Burden of Disease Study 2013. Lancet. 2015;386:743-800.
93 Naghavi M, Wang H, Lozano R, Davis A, Liang X, Zhou M, et al. Global, regional, and national age-sex specific all-cause and cause-specific mortality for 240 causes of death, 1990-2013: a systematic analysis for the Global Burden of Disease Study 2013. Lancet. 2015;385:117-71.
94 Lozano R, Naghavi M, Foreman K, Lim S, Shibuya K, Aboyans V, et al. Global and regional mortality from 235 causes of death for 20 age groups in 1990 and 2010: a systematic analysis for the Global Burden of Disease Study 2010. Lancet. 2012;380:2095-128.
95 Vos T, Flaxman AD, Naghavi M, Lozano R, Michaud C, Ezzati M, et al. Years lived with disability (YLDs) for 1160 sequelae of 289 diseases and injuries 1990-2010: a systematic analysis for the Global Burden of Disease Study 2010. Lancet. 2012;380:2163-96.
96 Rutten-van Molken MP, Postma MJ, Joore MA, Van Genugten ML, Leidl R, Jager JC. Current and future medical costs of asthma and chronic obstructive pulmonary disease in The Netherlands. Respir Med. 1999;93:779-87.
97 World Health Organization. The global burden of disease: 2004 update. Geneva: World Health Organization; 2008.
98 Mathers CD, Bernard C, Iburg KM, Inoue M, Ma Fat D, Shibuya K, et al. Global burden of disease in 2002: data sources, methods and results. Geneva: World Health Organization; 2003.
99 Mathers CD, Loncar D. Projections of global mortality and burden of disease from 2002 to 2030. PLoS Med. 2006;3:e442.
100 Mathers CD, Lopez AD, Murray CJL. The Burden of Disease and Mortality by Condition: Data, Methods, and Results for 2001. In: Lopez AD, Mathers CD, Ezzati M, Jamison DT, Murray CJL, editors. Global Burden of Disease and Risk Factors. Washington (DC): The World Bank; 2006. p. 45-93.
101 Mathers CD, Stein C, Ma Fat D, Rao C, Inoue M, Tomijima N, et al. Global Burden of Disease 2000: Version 2 methods and results. Geneva: World Health Organization; 2002.
102 Jarvis D, Newson R, Janson C, Corsico A, Heinrich J, Anto JM, et al. Prevalence of asthma-like symptoms with ageing. Thorax. 2018;73:37-48.
103 Cohen S, Berkman N, Avital A, Springer C, Kordoba L, Haklai Z, et al. Decline in asthma prevalence and severity in Israel over a 10-year period. Respiration. 2015;89:27-32.
104 Nurmagambetov T, Kuwahara R, Garbe P. The Economic Burden of Asthma in the United States, 2008-2013. Ann Am Thorac Soc. 2018;15:348-56.
105 Morris RD, Munasinghe RL. Geographic variability in hospital admission rates for respiratory disease among the elderly in the United States. Chest. 1994;106:1172-81.
106 Luyt DK, Burton PR, Simpson H. Epidemiological study of wheeze, doctor diagnosed asthma, and cough in preschool children in Leicestershire. BMJ. 1993;306:1386-90.
107 Goren AI, Hellmann S. Changing prevalence of asthma among schoolchildren in Israel. Eur Respir J. 1997;10:2279-84.

108 Aguinaga Ontoso I, Arnedo Pena A, Bellido J, Guillen Grima F, Suarez Varela MM. [The prevalence of asthma-related symptoms in 13-14-year-old children from 9 Spanish populations. The Spanish Group of the ISAAC Study (International Study of Asthma and Allergies in Childhood)]. Med Clin (Barc). 1999;112:171-5.Spanish.
